# Dual-target anti-Alzheimer’s disease agents with both iron ion chelating and monoamine oxidase-B inhibitory activity

**DOI:** 10.1080/14756366.2019.1634703

**Published:** 2019-08-16

**Authors:** Zhisheng Mi, Bing Gan, Sihang Yu, Jianan Guo, Changjun Zhang, Xiaoying Jiang, Tao Zhou, Jing Su, Renren Bai, Yuanyuan Xie

**Affiliations:** a College of Pharmaceutical Science, Zhejiang University of Technology, Hangzhou, China;; b Guiyang Institute for Food and Drug Control, Guiyang, China;; c Department of Pathophysiology, College of Basic Medical Sciences, Jilin University, Changchun, China;; d School of Food Science and Biotechnology, Zhejiang Gongshang University, Hangzhou, China;; e Collaborative Innovation Center of Yangtze River Delta Region Green Pharmaceuticals, Zhejiang University of Technology, Hangzhou, China

**Keywords:** Alzheimer’s disease, iron ion chelation, MAO-B, coumarin derivatives, hydroxypyridinone

## Abstract

MAO-B leads to an increase in the levels of hydrogen peroxide and oxidative free radicals, which contribute to the aetiology of the AD. Thus, both iron ion chelators and MAO-B inhibitors can be used to treat AD. Taking the coumarin derivatives and hydroxypyridinones as the lead compounds, a series of dual-target hybrids were designed and synthesised by Click Chemistry. The compounds were biologically evaluated for their iron ion chelating and MAO-B inhibitory activity. Most of the compounds displayed excellent iron ion chelating activity and moderate to good anti-MAO-B activity. Compounds **27b** and **27j** exhibited the most potent MAO-B inhibitory activity, with IC_50_ values of 0.68 and 0.86 μM, respectively. In summary, these dual-target compounds have the potential anti-AD activity.

## 1. Introduction

Alzheimer’s disease (AD) is a degenerative brain disease and the most common cause of dementia^1^. In AD, nerve cells in parts of the brain involved in cognitive function are damaged or destroyed, leading to difficulties in memory, language, problem-solving and other cognitive skills. People in the final stages of the disease are bed-bound and require around-the-clock care^2^. Metal ions are involved in several essential functions in the nervous system. For instance, iron ion is required to support the brain’s high respiratory rate as well as for myelination, gene expression and synthesis of neurotransmitters^3^. Furthermore, iron ion is involved in the pathophysiology of AD^4^. Iron ion is usually accumulated in the nervous system of patients with neurodegenerative diseases^3^. 2-amido-3-hydroxypyridin-4-one, a chelating pharmacophore, was reported to show potential as non-toxic therapeutic agents for the treatment of AD^5^^,^^6^. Therefore, iron ion chelation can be a potential strategy for the AD treatment.

Monoamine oxidases (MAOs) are flavin adenine dinucleotide containing enzymes, which exist as two different isoforms, MAO-A and MAO-B. MAO-A is an effective target for the development of antidepressive drugs, while MAO-B was able to lead to an increase in the levels of hydrogen peroxide and oxidative free radicals, which contribute to the aetiology of the AD. Thus, selective MAO-B inhibitors can be used to treat neurodegenerative disorders such as AD^7^. Natural products have been an exemplary source of new drugs, and many of the currently available medicines have been directly or indirectly derived from them^8^. Coumarins are common natural products, showing a variety of pharmacological activities such as antithrombotic, anti-inflammatory, antihypertension, antitumor, and anti-AD effects^9^^,^^10^. Some coumarin derivatives were reported to demonstrate MAO-B inhibitory activity, which is contributed to their potential anti-AD activity^11^. All in all, coumarin is an optional scaffold to design MAO-B inhibitors for the treatment of AD.

**Figure 1.**
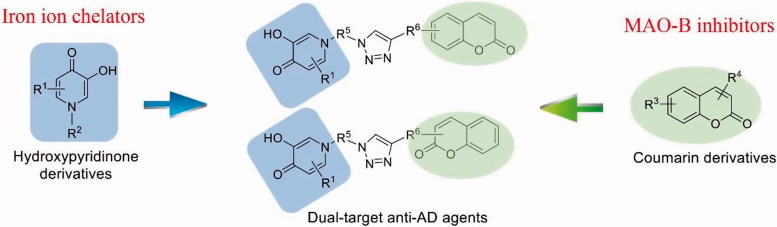
Strategy for the design of dual-target anti-AD agents.

Clearly, therapy with a single drug that has multiple biological properties is an effective strategy in the discovery of potent anti-AD lead compounds^12–14^. If the iron ion chelating and the MAO-B inhibitory pharmacophores were fused in one molecule, the hybrid might show more potent anti-AD activity. To obtain dual-target anti-AD agents, the structures of iron ion chelating hydroxypyridinones derivatives and the coumarin derivatives were combined *via* Click Chemistry (Figure 1). A series of dual-target compounds were designed, synthesised and biologically evaluated.

## 2. Material and methods

### 2.1. Chemistry

#### 2.1.1. Procedure for the synthesis of intermediate 2

Dry potassium carbonate (58.5 mmol), ethyl maltol (35.7 mmol), and iodomethane (179.0 mmol) were dissolved in acetone (100 mL). After refluxing for 4 h, the reaction mixture was concentrated. Water (200 mL) was added, and the mixture was extracted with dichloromethane (3 × 100 mL). The combined organic layer was washed with brine, dried over anhydrous sodium sulphate and the solvent was removed under reduced pressure to afford intermediate **2** as a yellow oil^15^.

#### 2.1.2. General procedures for the synthesis of intermediates 3, 4, 11, 12, 17, and 18

A mixture of intermediate **2** (23.2 mmol), amine R-NH_2_ (25.5 mmol), sodium hydroxide (50.0 mmol) in methanol/water (20 ml/20 mL) was refluxed. The reaction was monitored by TLC. After completion of the reaction (about 12 h), the reactant was concentrated under reduced pressure to about half volume. After extracting with dichloromethane (3 × 40 mL), the combined organic layers were washed with brine twice and dried over anhydrous sodium sulphate. After removal of the solvent, the crude product **3** was obtained as a brown oil^16^.

#### 2.1.3. General procedures for the synthesis of intermediates 5, 6, 13, 14, 19, and 20

To a mixture of compound **3** (7.4 mmol), triphenylphosphine (8.2 mmol), and sodium azide (37.0 mmol) in dry DMF (50 mL) was added to carbon tetrabromide (8.2 mmol) at room temperature. The mixture was stirred at room temperature for 1 h, the residue was purified by short-column chromatography using a gradient of MeOH in dichloromethane (3–5%) to yield **5**^17^.

#### 2.1.4. Procedure for the synthesis of intermediate 8

Kojic acid (**7**) (1.4 mol) was dissolved in thionyl chloride (800 mL) and stirred for 1 h. The product was collected by filtration and washed with petroleum ether, then recrystallized from water to yield a white solid. The white solid intermediate (0.9 mol) was added to 500 mL of distilled water and heated to 50 °C with stirring. Zinc dust (1.9 mol) was added followed by the dropwise addition of concentrated hydrochloric acid (280 mL) over 1 h with vigorous stirring at the temperature between 70 and 80 °C. The reaction mixture was stirred for a further 3 h at 70 °C. Excess zinc was removed by hot filtration and the filtrate extracted with dichloromethane (10 × 200 mL). The combined organic extracts were dried, filtered, and concentrated in vacuum to provide the crude product. Recrystallization from isopropanol afforded compound **8**^18^.

#### 2.1.5. Procedure for the synthesis of intermediate 9

6-Methyl-3-hydroxypyran-4(1H)-one (**8**) (54.0 mmol) was added to an aqueous solution of sodium hydroxide (59.4 mmol) in distilled water (50 mL) and stirred at room temperature for 5 min. 35% Formaldehyde solution (5 mL) was added dropwise over 10 min and the solution was allowed to stir for 12 h. After acidification of the reaction mixture to pH = 1 using concentrated hydrochloric acid and cooling to 3–5 °C for 12 h, intermediate **9** was obtained. ^1^H NMR (600 MHz, DMSO-d_6_) δ 6.22 (s, 1H), 4.39 (d, J = 6 Hz, 2H), 2.27 (s, 3H).

#### 2.1.6. Procedure for the synthesis of intermediate 10

Intermediate **9** (3.7 mmol) was added to DMSO (30 mL) and the mixture was stirred until intermediate **9** was completely dissolved. This solution was added to KOH (7.3 mmol) and stirred for 3 h. The above reaction vessel was sealed and iodomethane (18.3 mmol) was added dropwise over 10 min and the reaction mixture was allowed to stir overnight. A reddish brown clear mixture was obtained at the end of the period. The reaction mixture was concentrated under pressure and the solid was dissolved in 500 mL water. The aqueous layer was extracted with DCM (5 × 100 mL). The combined extracts was filtered and dried to obtain the crude product as yellow oil. Column chromatography using MeOH/CHCl_3_ (1: 20, V/V) yielded the pure compound **10** as a white solid^18^.

#### 2.1.7. Procedure for the synthesis of intermediate 15

Intermediates **9** (35.7 mmol), dried potassium carbonate (58.5 mmol) and iodomethane (179 mmol) were dissolved in acetone (100 mL). After refluxing for 4 h, the reaction mixture was concentrated. Water (200 mL) was added and extracted with CH_2_Cl_2_ (3 × 100 mL). The combined organic layers were washed with brine, dried over MgSO_4,_ and the solvent was removed under reduced pressure to afford **15** as a yellow oil. The crude product was purified by column chromatography with DCM/methanol (150:1, V/V) to yield the product as a yellow oil^15^.

#### 2.1.8. Procedure for the synthesis of intermediate 16

A solution of intermediate **15** (10.0 mmol) in DCM (20 mL) was treated with thionyl chloride (12.0 mmol) at room temperature. The resulting mixture was stirred for 1 h. Then, methanol (10 mL) was added to the mixture. After removal of the solvent, the crude intermediate was obtained as a brown oil. The intermediate (5.0 mmol) was then added to 10 ml of distilled water and the mixture was heated to 50 °C with stirring. Zinc dust (10 mol) was added followed by the dropwise addition of concentrated hydrochloric acid (5 mL) over 1 h with vigorous stirring at the temperature between 70 and 80 °C. The reaction mixture was stirred for a further 3 h at 70 °C. After removal of the solvent, the crude intermediate **16** was obtained as a yellow oil^18^.

#### 2.1.9. General procedure for the synthesis of intermediates 22, 24, and 26

Propargyl bromide (2.4 mmol) was added to a suspension of the coumarin (2.0 mmol) and potassium carbonate (4.0 mmol) in acetone (15 mL). The suspension was heated under reflux for 2 h. The reaction was allowed to cool and then concentrated under reduced pressure. Water (30 mL) was added and extracted with EtOAc (3 × 30 mL). The organic extracts were combined and washed with water (20 mL) and brine (20 mL) dried over anhydrous sodium sulphate, then filtered and concentrated *in vacuo*. The crude product was purified by column chromatography^18^.

#### 2.1.10. General procedures for the synthesis of target compounds 27a-k

To a stirred solution of intermediate **13** (2.0 mmol) and alkyne **24** (2.2 mmol) in MeOH/H_2_O (3:3, V/V), CuSO_4_.5H_2_O (2.2 mmol) and sodium ascorbate (0.5 mmol) were added. The reaction mixture was stirred at room temperature for 2 h and then concentrated under reduced pressure. Water (10 mL) was added and the mixture was extracted with dichloromethane (3 × 20 mL). The combined organic phases were washed over water (20 mL), dried over anhydrous sodium sulphate, and evaporated to dryness. The crude was purified by column chromatography with dichloromethane and methanol (150:1, v/v) to yield the products as white solids. Then, BBr_3_ (1.5 mmol) was added dropwise to a solution of this white solid (0.5 mmol) in dichloromethane (10 mL) at 0 °C under N_2_ atmosphere. The reaction mixture was then warmed up to room temperature and the stirring continued for 12 h. After the reaction, methanol (10 mL) was added to the mixture and was stirred for another 0.5 h. Concentration and recrystallization from methanol/diethyl ether gave compounds ***27a-k*** as white solids (supplementary material)^19^^,^^20^.

##### 2.1.10.1. 3-hydroxy-2-(hydroxymethyl)-6-methyl-1–(2-(4-(((2-oxo-2H-chromen-7-yl) oxy)methyl)-1H-1,2,3-triazol-1-yl)ethyl)pyridin-4(1H)-one (27a)

yellow solid, yield: 81%; m.p.: 212–215 °C; ^1^H NMR (600 MHz, DMSO-d_6_) δ 8.34 (s, 1H), 8.02 (d, *J* = 9.5 Hz, 1H), 8.01 (d, *J* = 8.7 Hz, 1H), 7.66 (d, *J* = 2.5 Hz, 1H), 7.25 (s, 1H), 7.01 (dd, *J* = 8.6, 2.4 Hz, 1H), 6.30 (d, *J* = 9.5 Hz, 1H), 5.29 (s, 2H), 4.99 (d, *J* = 5.9 Hz, 2H), 4.93 (s, *J* = 5.9 Hz, 2H), 4.62 (s, 2H), 2.37 (s, 3H). ^13 ^C NMR (150 MHz, DMSO-d_6_) δ 161.5, 161.4, 160.7, 155.7, 149.2, 144.7, 142.9, 142.1, 142.0, 130.0, 126.3, 113.5, 113.3, 113.2, 113.1, 102.1, 61.9, 53.2, 50.3, 48.5, 20.1. HRMS (ESI) calcd for C_21_H_21_N_4_O_6_ [M + H]^+^: 425.1456; found: 425.1439.

##### 2.1.10.2. 3-hydroxy-2-(hydroxymethyl)-6-methyl-1–(3-(4-(((2-oxo-2H-chromen-7-yl)oxy)methyl)-1H-1,2,3-triazol-1-yl)propyl)pyridin-4(1H)-one (27b)

yellow solid, yield: 84%; m.p.: 189–192 °C; ^1^H NMR (600 MHz, DMSO-d_6_) δ 8.41 (s, 1H), 8.01 (d, *J* = 9.5 Hz, 1H), 7.66 (d, *J* = 8.6 Hz, 1H), 7.15 (s, 1H), 7.12 (s, 1H), 7.02 (dd, *J* = 8.5 Hz, 1H), 6.30 (d, *J* = 9.4, 1.7 Hz, 1H), 5.29 (s, 2H), 4.76 (s, 2H), 4.61 (t, *J* = 6.7 Hz, 2H), 4.45 (t, *J* = 8.5 Hz, 2H), 2.38 (s, 3H), 2.37 (m, 6.9 Hz, 2H). ^13 ^C NMR (150 MHz, DMSO-d_6_) δ 163.5, 161.5, 160.7, 160.0, 155.7, 148.8, 144.7, 142.7, 142.5, 142.3, 130.0, 125.5, 113.5, 113.3, 113.0, 102.0. 62.1. 53.3. 48.7. 47.2 30.0 20.0. HRMS (ESI) calcd for C_22_H_23_N_4_O_6_ [M + H]^+^: 439.1612; found: 439.1606.

##### 2.1.10.3. 3-hydroxy-2,6-dimethyl-1–(2-(4-(((2-oxo-2H-chromen-7-yl)oxy)methyl)-1H-1,2,3-triazol-1-yl)ethyl)pyridin-4(1H)-one (27c)

white solid, yield: 93%; m.p.: 256–249 °C; ^1^H NMR (600 MHz, DMSO-d_6_) δ 8.34 (s, 1H), 8.02 (d, *J* = 9.5 Hz, 1H), 7.66 (d, *J* = 8.6 Hz, 1H), 7.14 (s, 1H), 7.03 (s, 1H), 7.01 (dd, *J* = 8.6, 2.5 Hz, 1H), 6.32 (d, *J* = 9.5 Hz, 1H), 5.30 (s, 2H), 4.93 (t, *J* = 6.3 Hz, 2H), 4.80 (t, *J* = 6.2 Hz, 2H), 2.41 (s, 3H), 2.34 (s, 3H). ^13 ^C NMR (150 MHz, DMSO-d_6_) δ 161.4, 160.7, 159.1, 155.7, 148.4, 144.8, 143.1, 142.9, 141.9, 130.0, 126.4, 113.4, 113.2, 113.1, 112.6, 102.1, 61.9, 51.1, 47.7, 20.3, 13.6. HRMS (ESI) calcd for C_21_H_21_N_4_O_5_ [M + H]^+^: 409.1518; found: 409.1506.

##### 2.1.10.4. 3-hydroxy-2,6-dimethyl-1–(3-(4-(((2-oxo-2H-chromen-7-yl)oxy)methyl)-1H-1,2,3-triazol-1-yl)propyl)pyridin-4(1H)-one (27d)

white solid, yield: 87%; m.p.: 263–265 °C; ^1^H NMR (600 MHz, DMSO-d_6_) δ 8.43 (s, 1H), 8.01 (d, *J* = 9.4 Hz, 1H), 7.66 (d, *J* = 8.5 Hz, 1H), 7.15 (s, 1H), 7.06 (s, 1H), 7.02 (d, *J* = 8.7 Hz, 1H), 6.30 (d, *J* = 9.4 Hz, 1H), 5.30 (s, 2H), 4.62 (t, *J* = 6.7 Hz, 2H), 4.28 (t, *J* = 6.7 Hz, 2H), 2.51 (s, *J* = 7.0 Hz, 5H), 2.47 (s, 3H). ^13 ^C NMR (150 MHz, DMSO-d_6_) δ 161.5, 160.7, 158.6, 155.7, 147.6, 144.7, 142.6, 142.5, 141.8, 130.0, 125.6, 113.4, 113.1, 113.1, 112.6, 102.1, 62.2, 49.1, 47.1, 28.8, 20.3, 13.4. HRMS (ESI) calcd for C_22_H_23_N_4_O_5_ [M + H]^+^: 423.1663; found: 423.1676.

##### 2.1.10.5. 2-ethyl-3-hydroxy-1–(3-(4-(((2-oxo-2H-chromen-7-yl)oxy)methyl)-1H-1,2,3-triazol-1-yl)propyl)pyridin-4(1H)-one (27e)

white solid, yield: 91%; m.p.: 204–206 °C; ^1^H NMR (600 MHz, DMSO-d_6_) δ 8.29 (s, 1H), 8.02 (d, *J* = 9.5 Hz, 1H), 7.88 (d, *J* = 7.1 Hz, 1H), 7.66 (d, *J* = 8.6 Hz, 1H), 7.13 (d, *J* = 2.4 Hz, 1H), 7.05 (d, *J* = 6.6 Hz, 1H), 7.00 (dd, *J* = 8.6, 2.4 Hz, 1H), 6.32 (d, *J* = 9.5 Hz, 1H), 5.27 (s, 2H), 4.47 (s, 2H), 3.95 (s, 2H), 2.58 (s, 2H), 2.24 (s, 2H), 1.02 (s, 3H). ^13 ^C NMR (150 MHz, DMSO-d_6_) δ 169.7, 161.5, 160.7, 155.8, 144.8, 142.6, 137.8, 137.7, 134.1, 129.9, 125.5, 113.4, 113.3, 113.0, 102.0, 62.1, 55.1, 49.8, 47.1, 31.9, 18.9, 13.0.HRMS (ESI) calcd for C_21_H_21_N_4_O_5_ [M + H]^+^: 409.1506; found: 409.1512.

##### 2.1.10.6. 2-ethyl-3-hydroxy-1–(2-(4-(((2-oxo-2H-chromen-4-yl)oxy)methyl)-1H-1,2,3-triazol-1-yl)ethyl)pyridin-4(1H)-one (27f)

White solid, yield: 90%; m.p.: 253–256 °C ^1^H NMR (600 MHz, DMSO-d_6_) δ 8.28 (s, 1H), 8.01 (d, *J* = 7.0 Hz, 1H), 7.87 (d, *J* = 7.8 Hz, 1H), 7.65 (d, *J* = 8.4, 7.2, Hz, 1H), 7.12 (d, *J* = 8.4 Hz, 1H), 7.00 (d, *J* = 7.2 Hz, 1H), 6.99 (d, *J* = 7.2 Hz, 1H), 6.31 (d, H), 5.27 (s, 2H), 4.91 (t, *J* = 6.9 Hz, 2H), 4.85 (t, *J* = 6.9 Hz, 2H), 2.68 (q, *J* = 6.0 Hz, 2H), 1.08 (t, *J* = 6.0 Hz, 3H). ^13 ^C NMR (150 MHz, DMSO-d_6_) δ 161.4, 160.7, 159.3, 155.7, 146.9, 144.8, 143.2, 142.8, 138.8, 130.1, 126.3, 113.3, 113.2, 113.1, 111.3, 102.1, 61.9, 55.1, 49.8, 19.8, 12.1. HRMS (ESI) calcd for C_21_H_21_N_4_O_5_ [M + H]^+^: 409.1506; found: 409.1500.

##### 2.1.10.7. 2-ethyl-3-hydroxy-1–(3-(4-(((2-oxo-2H-chromen-4-yl)oxy)methyl)-1H-1,2,3-triazol-1-yl)propyl)pyridin-4(1H)-one (27g)

White solid, yield: 92%; m.p.: 195–198 °C; ^1^H NMR (600 MHz, DMSO-d_6_) δ 8.48 (s, 1H), 8.29 (d, *J* = 7.0 Hz, 1H), 7.74 (d, *J* = 7.8 Hz, 1H), 7.66 (t, *J* = 8.0 Hz, 1H), 7.41 (d, *J* = 8.4 Hz, 1H), 7.33 (t, *J* = 7.2 Hz, 1H), 7.20 (d, *J* = 7.2 Hz, 1H), 6.17 (s, 1H), 5.45 (s, 2H), 4.59 (t, *J* = 6.6 Hz, 2H), 4.43 (t, *J* = 6.6 Hz, 2H), 2.87 (q, *J* = 7.5 Hz, 2H), 2.41 (m, 2H), 1.12 (t, *J* = 7.2 Hz, 3H). ^13 ^C NMR (150 MHz, DMSO-d_6_) δ 164.7, 161.9, 159.1, 153.2, 146.5, 143.3, 141.6, 138.8, 133.2, 125.7, 124.6, 123.3, 117.1, 115.6, 111.7, 91.8, 63.2, 53.2, 47.0, 31.5, 20.2, 12.2. HRMS (ESI) calcd for C_22_H_23_N_4_O_5_ [M + H]^+^: 423.1815; found: 423.1826.

##### 2.1.10.8. 3-hydroxy-2-(hydroxymethyl)-6-methyl-1–(2-(4-(((2-oxo-2H-chromen-4-yl)oxy)methyl)-1H-1,2,3-triazol-1-yl)ethyl)pyridin-4(1H)-one (27h)

White solid, yield: 89%; m.p.: 142–148 °C;^1^H NMR (600 MHz, DMSO-d_6_) δ 8.45 (s, 1H), 7,73 (d, *J* = 8.4 Hz, 1H), 7.68 (d, *J* = 8.4 Hz, 1H), 7.40 (m, 2H), 7.26 (s, 1H), 6.17 (s, *J* = 8.4 Hz, 1H), (s, 1H), 5.46 (s, 2H), 5.04 (d, *J* = 6.0 Hz, 2H), 4.98 (d, *J* = 6.0 Hz, 2H), 4.64 (s, 2H), 3.19 (s, 3H), 2.44(s, 3H). ^13 ^C NMR (150 MHz, DMSO-d_6_) δ 164.7, 161.9, 161.7, 153.2, 149.3, 143.0, 141.9, 141.8, 143.3, 126.7, 124.6, 123.4, 117.0, 115.5, 113.6, 91.8, 63.1, 53.2, 50.3, 49.1, 20.1. HRMS (ESI) calcd for C_21_H_21_N_4_O_6_ [M + H]^+^: 425.1415; found: 425.1418.

##### 2.1.10.9. 2-ethyl-3-hydroxy-1–(2-(4-(((2-oxo-2H-chromen-3-yl)oxy)methyl)-1H-1,2,3-triazol-1-yl)ethyl)pyridin-4(1H)-one (27i)

White solid, yield: 88%; m.p.: 258–261 °C; ^1^H NMR (600 MHz, DMSO-d_6_) δ 8.40 (s, 1H), 7.93 (d, *J* = 7.0 Hz, 1H), 7.72 (d, *J* = 7.9 Hz, 1H), 7.68 (t, *J* = 7.6 Hz, 1H), 7.42 (d, *J* = 8.4 Hz, 1H), 7.38 (t, *J* = 7.6 Hz, 1H), 7.08 (d, *J* = 6.9 Hz, 1H), 6.12 (s, 1H), 5.43 (s, 2H), 4.96 (t, *J* = 6 Hz, 2H), 4.89 (t, *J* = 6 Hz, 2H), 2.73 (q, *J* = 7.8 Hz, 2H), 1.10 (t, *J* = 7.4 Hz, 3H). NMR (150 MHz, DMSO-d_6_) δ 160.3, 160.1, 159.2, 154.1, 152.4, 147.5, 143.2, 142.0, 130.8, 125.6, 124.9, 113.5, 113.0, 111.8, 110.9, 101.4, 61.4, 54.1, 48.2, 18.0, 17.2 HRMS (ESI) calcd for C_21_H_21_N_4_O_5_ [M + H]^+^: 409.1612; found: 409.1607.

##### 2.1.10.10. 2-ethyl-3-hydroxy-1–(3-(4-(((2-oxo-2H-chromen-3-yl)oxy)methyl)-1H-1,2,3-triazol-1-yl)propyl)pyridin-4(1H)-one (27j)

White solid, yield: 90%; m.p.: 192–195 °C; ^1^H NMR (600 MHz, DMSO-d_6_) δ 8.46 (s, 1H), 8.27 (d, *J* = 7.0 Hz, 1H), 7.75 (d, *J* = 7.9, 1.6 Hz, 1H), 7.67 (t, *J* = 8.6, 7.2, Hz, 1H), 7.42 (d, *J* = 8.3 Hz, 1H), 7.35 (t, *J* = 7.1 Hz, 1H), 7.18 (d, *J* = 7.0 Hz, 1H), 6.18 (s, 1H), 5.45 (s, 2H), 4.58 (t, *J* = 6.9 Hz, 2H), 4.42 (t, *J* = 6.9 Hz, 2H), 2.87 (q, *J* = 7.5 Hz, 2H), 2.41 (m, 2H), 1.17 (t, *J* = 7.5 Hz, 3H). ^13 ^C NMR (150 MHz, DMSO-d_6_) δ 166.8, 160.4, 159.3, 152.2, 147.4, 145.8, 143.2, 142.1, 130.6, 130.6, 125.4, 124.7, 117.7, 113.5, 111.8, 101.2, 61.4, 53.9, 48.2, 17.9, 14.3, 12.4. HRMS (ESI) calcd for C_22_H_23_N_4_O_5_ [M + H]^+^: 423.1653; found: 423.1609.

##### 2.1.10.11. 3-hydroxy-2-(hydroxymethyl)-6-methyl-1–(3-(4-(((2-oxo-2H-chromen-3-yl)oxy)methyl)-1H-1,2,3-triazol-1-yl)propyl)pyridin-4(1H)-one (27k)

White solid, yield: 85%; m.p.: 168–170 °C; ^1^H NMR (600 MHz, DMSO-d_6_) δ 8.49 (s, 1H), 7.76 (d, *J* = 8.0 Hz, 1H), 7.67 (t, *J* = 7.2 Hz, 1H), 7.42 (d, *J* = 7.2 Hz, 1H), 7.35 (t, *J* = 7.8 Hz, 1H), 7.11 (s, 1H), 6.17 (s, 1H), 5.46 (s, 2H), 4.76 (s, 2H), 4.63 (t, *J* = 6.6 Hz, 2H), 4.46 (s, 2H), 2.55 (s, 3H), 2.40 (m, 2H). ^13 ^C NMR (150 MHz, DMSO-d_6_) δ 163.3, 161.5, 160.7, 159.8, 155.7, 148.8, 144.7, 142.7, 142.5, 142.3, 129.8, 125.5, 113.5, 113.3, 112.9, 102.0. 62.1. 53.1. 48.7. 47.2 30.0 19.8. HRMS (ESI) calcd for C_22_H_23_N_4_O_6_ [M + H]^+^: 439.1616; found: 439.1603.

### 2.2. Iron ion chelating activity determination

#### 2.2.1. Instruments and reagents

The automatic titration system used in the test consisted of an automatic titrator, a luminescence 759 s UV-Vis spectrophotometer, and a pH Meter (Mettler Toledo InLab Expert PRO/Mettler Toledo InLab Science). All instruments are controlled by a computer program.

The testing solution (2:3 DMSO/KCl (0.1 M), V/V) was maintained at 25 °C using a TP-3A temperature controller. The cuvette is mounted on a spectrophotometer. The E0 and s of the pH Meter were calibrated by three standard solutions (pH = 6.86, 9.18, 4.00). KCl solution (0.1 M) was used to maintain the ionic strength. For the Fe(III) affinity evaluation, the ratio of compounds to iron ion was kept at 1.1:1 (n/n) when Logβ_1_ was measured; the ratio of compounds to iron ion was kept at 5:1 (n/n) when Logβ_2_ and Logβ_3_ were tested.

#### 2.2.2. p*K*_a_ determination

The titration was performed in a 50 mm path length cuvette. 45 ml KCl (0.1 M) solution, 40 μL of saturated EDTA solution were added to the cuvette. The pH value of the solution was acidified to 2.0 with hydrochloric acid (1.5 M). Then 20 μL of test compound DMSO solution (30 μM/L) was added and stirred vigorously. By adding KOH solution (0.1 M) from the automatic buret, the pH value of the solution was increased by 0.1. After the system was equilibrated (when the change of pH value did not exceed 0.001 within 3 s), the spectral scan was performed once after 30 s. Adding KOH solution (0.1 M) and repeated the automatic process until the specified endpoint of pH value was reached. All data were recorded internally by the Visual Basic program, and the test results are analysed by the HypSpec2014 program^21^.

#### 2.2.3. Determination of iron(III) affinity

##### 2.2.3.1. Determination of Logβ_1_

A 45 ml KCl (0.1 M) solution was added to the cuvette (50 mm) and the pH value was acidified to 2.1. Then 30 μM DMSO solution of the testing compound and 60 μL acidic solution of FeCl_3_ (15 mM) were added separately, and the mixture was vigorously stirred. After the absorbance value is stabilised, a certain amount of HCl solution (4 M) was added by the automatic buret to reduce the pH of the solution by 0.1. After the system was equilibrated, the spectral scan was performed once after 20 min. This protocol was repeated until the specified endpoint of the pH value was reached. All data was recorded with the internal Visual Basic program, HypSpec2014 program was utilised for the data analysis^21^.

##### 2.2.3.2. Determination of Logβ_2_ and Logβ_3_

Similarly, 45 ml KCl (0.1 M) solution was added to the cuvette (50 mm) and the pH value was acidified to 2.1. Then 30 μM DMSO solution of the testing compound and 60 μL acidic solution of FeCl_3_ (15 mM) were added separately, and the mixture was vigorously stirred. After the absorbance value is stabilised, a certain amount of KOH solution (0.1 M) was added by the automatic buret to increase the pH of the solution by 0.1. After the system was equilibrated, the spectral scan was performed once after 5 min. This protocol was repeated until the specified endpoint of the pH value was reached. All data were recorded with the internal Visual Basic program, HypSpec2014 program was utilised for the data analysis^5^^,^^21^^,^^22^.

#### 2.2.4. Calculation of pFe^3+^

The values of pFe^3+^ were calculated by HySS software according to the results of p*K*_a1_, p*K*_a2_, logβ_1_, logβ_2_, and logβ_3_ determined by the above methods. The basic parameters were set as follows: FeOH = –2.563, Fe(OH)_2_ = –6.205, Fe(OH)_3_ = –15.1, Fe_2_(OH)_2_ = –2.843, Fe_3_(OH)_4_ = –6.059, Fe(OH)_4_ = –21.883^23^.

### 2.3. Determination of human MAO-B inhibitory activity

Human MAO-B (5 mg/mL) was purchased from Sigma-Aldrich and was pre-aliquoted and stored at -70 °C. 200 μL enzymatic reactions were diluted in 2 ml microcentrifuge tubes by potassium phosphate buffer (Assay Buffer, pH 7.4) which have been provided by Sigma-Aldrich Monoamine Oxidase Assay Kit. The final volumes of the reactions were 100 μL and contained 45 μL MAO-B (0.0075 mg/mL), included 5 μL different concentrations of the testing inhibitors (10 μM, 1 μM), and 50 μL Master Reaction, p-Tyramine, HRP Enzyme and Dye Reagent. Stock solutions of the compounds were prepared in DMSO. The reactions were incubated for 15 min at 37 °C in a flat-black bottom 96-well micro test plate in the dark. The results were quantified in a multi-detection microplate fluorescence reader based on the fluorescence generated (excitation, 535 nm; emission, 585 nm). To calculate the IC_50_ values, these data were fitted to the one site competition model incorporated into the Prism software package (GraphPad)^24^^,^^25^.

## 3. Results and discussion

### 3.1. Chemistry

**Scheme 1.**
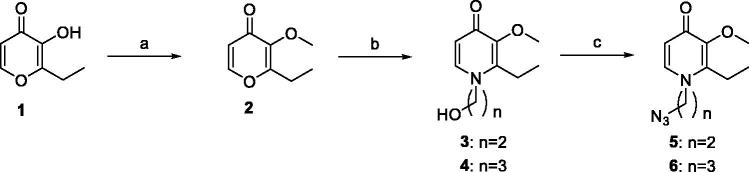
The synthetic route of intermediates **5** and **6**. Reagents and conditions: (a) Acetone, CH_3_I, K_2_CO_3,_ r.t., 4 h, 93%; (b) MeOH:H_2_O = 1:1, NH_2_(CH_2_)*_n_*OH, NaOH, reflux, 12 h, 81%–83%; (b) DMF, NaN_3_, Ph_3_P, CBr_4_, r.t., 1 h, 75%–86%.

**Scheme 2.**
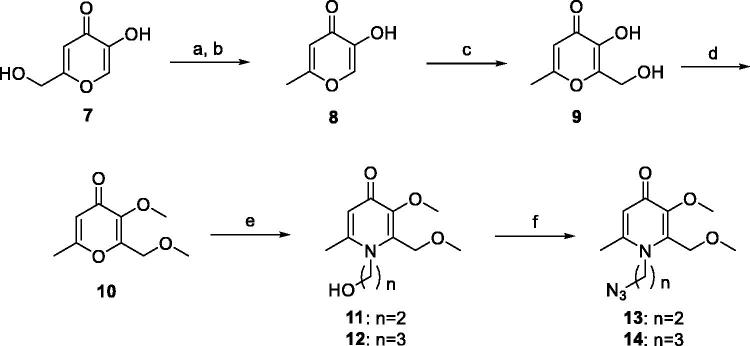
The synthetic route of intermediates **13** and **14**. Reagents and conditions: (a) SOCl_2_, r.t., 4 h, 87%; (b) H_2_O, Zn, HCl, 70 °C, 72%; (c) 2.0 M NaOH, 37% HCHO, r.t., 12 h, 80%; (d) DMSO, CH_3_I, KOH_,_ r.t., 10 h, 92%; (e) MeOH:H_2_O = 1:1, NH_2_(CH_2_)*_n_*OH, NaOH, reflux, 12 h, 78%–82%; (f) DMF, NaN_3_, Ph_3_P, CBr_4_, r.t., 1 h, 76%–78%.

**Scheme 3.**
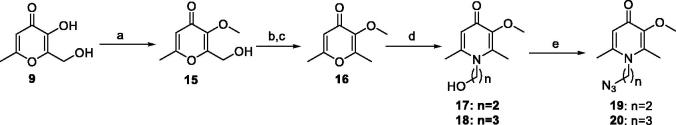
The synthetic route of intermediates **19** and **20**. Reagents and conditions: (a) Acetone, CH_3_I, K_2_CO_3,_ r.t., 4 h, 93%; (b) CH_2_Cl_2_, SOCl_2_, r.t., 4 h, 87%; (c) H_2_O, Zn, HCl, 70 °C, 72%; (d) MeOH:H_2_O = 1:1, NH_2_(CH_2_)*_n_*OH, NaOH, reflux, 12 h, 77%–79%; (e) DMF, NaN_3_, Ph_3_P, CBr_4_, r.t., 1 h, 79%–81%.

**Scheme 4.**
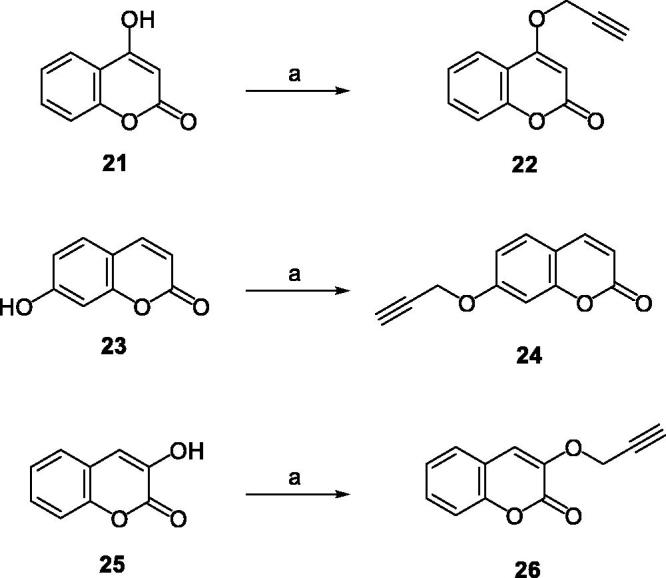
The synthetic route of intermediates **22, 24** and **26**. Reagents and conditions: (a) Acetone, Propargyl bromide, K_2_CO_3,_ reflux, 2 h, 74%–86%.

**Scheme 5.**
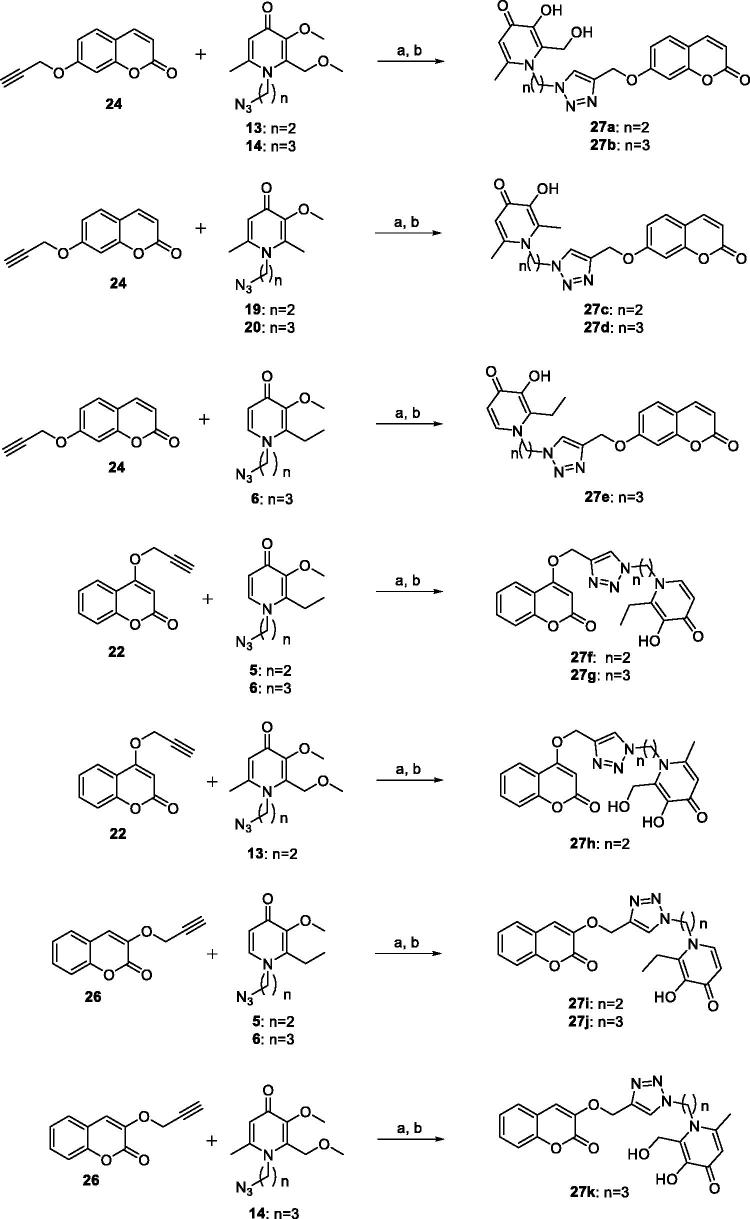
The synthetic route of compounds **27a-k**. Reagents and conditions: (a) MeOH:H_2_O = 1:1, CuSO_4_·5H_2_O, sodium ascorbate, 25 °C, 2 h, 81%–92%; (b) CH_2_Cl_2_, BBr_3_, MeOH, 0 °C- r.t., 1 h, 87%–95%.

The synthetic routes of coumarin–pyridinone hybrids **27a–k** were outlined in Schemes 1–5. Kojic acid (**7**) was used as starting material for the preparation of intermediates **13** and **14**. The hydroxymethyl moiety was substituted with the chloromethyl group, which was subsequently reduced by zinc/hydrochloric acid, and the resulting compound **8** was subjected to aldol condensation reaction to introduce a hydroxymethyl moiety to the pyridone scaffold. Protection of the hydroxyl group was achieved by methylation using iodomethane in the presence of K_2_CO_3_ or KOH. The oxygen atom was substituted with the nitrogen *via* reaction with appropriate primary amines to provide the corresponding HPO derivatives **11** and **12** in modest yields. Subsequently, Mitsunobu Reaction was used to synthesised intermediates **13** and **14**. Compounds **5**, **6**, **19**, **20** were prepared under similar conditions to the synthesis of compounds **13** and **14**. Key intermediates **22**, **24**, and **26** were efficiently prepared by reaction of propargyl bromide with appropriate commercially available reagents **21**, **23**, and **25**. Finally, designed compounds **27a-k** were obtained through the Huisgen 1, 3-Dipolar Cycloaddition Reaction (Click Chemistry) of alkyne intermediates **22**, **24**, and **26** and corresponding azides, followed by demethylation with BBr_3_.

### 3.2. Iron ion chelating activity

**Scheme 6.**
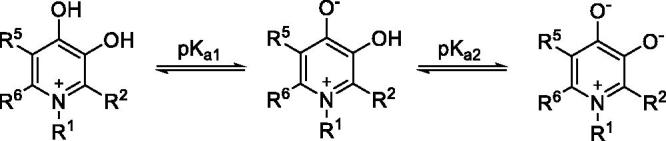
The ionisation of hydroxypyridones.

3-Hydroxypyridin-4-one usually has two p*K*_a_ values. The p*K*_a1_ is assigned to the protonation of the 4-oxo group and p*K*_a2_ is assigned to the dissociation of the 3-hydroxy group (Scheme 6).

**Figure 2.**
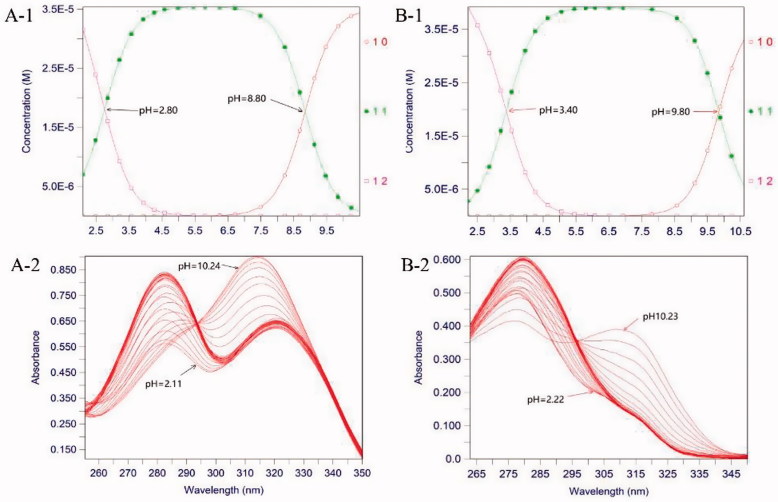
The pH-dependent UV spectra of compounds **27b** and **27f**. **A-1**. The p*K*_a_ values of compound **27b**. **A-2**. The pH-dependence of the spectrum of compound **27b** over the pH range 2.11 and 10.24 in 0.1 M KCl at 25 ^o^C. **B-1**. The p*K*_a_ values of compound **27j**. **B-2**. The pH-dependence of the spectrum of compound **27j** over the pH range 2.22 and 10.23 in 0.1 M KCl at 25 °C.

The p*K*_a_ values and Fe^3+^ chelating activity of all compounds were listed in Table 1. For free ligands (compounds), the pH-dependent UV spectra of the series of compounds were recorded between 255 and 350 nm in the pH range of 2.4–11.0. The spectroscopy displayed a significant change of the maximum from 280 nm to 315 nm, which reflected the pH-dependence of compounds ionisation equilibrium. The two p*K*_a_ values of the ligand were shown in Figure 2.

**Table 1.** The p*K*_a_ and pFe^3+^ values of the compounds **27a-k**^a^.

Compound	p*K*_a1_	p*K*_a2_	Logβ_1_	Logβ_2_	Logβ_3_	pFe^3+^
**27a**	2.7	9.2	13.8	24.0	33.7	18.9
**27b**	2.8	8.8	12.9	25.5	33.5	19.8
**27c**	3.3	9.7	14.9	26.0	34.1	18.0
**27d**	3.4	10.0	14.9	26.3	34.8	17.6
**28e**	3.2	9.8	14.7	26.0	33.2	17.1
**27f**	3.4	9.8	14.9	25.7	32.8	16.7
**27g**	3.2	9.7	14.6	25.6	33.1	17.1
**27h**	3.0	9.1	13.3	24.8	33.2	18.7
**27i**	2.9	9.6	14.2	25.4	32.8	17.0
**27j**	3.3	9.8	14.6	25.7	33.5	17.1
**27k**	3.3	9.1	13.6	24.5	33.0	18.4
Deferiprone	3.68	9.70	14.83	25.7	33.6	17.4
				27.0^b^	36.4^b^	20.1^b^
Deferiprone	3.61^c^	9.78^c^	15.03^c^	27.42^c^	37.35^c^	20.7^c^

a The compounds were tested in DMSO:KCl (0.1 M) = 2:3 (V/V) to address the solubility issue.

b The data was measured in 0.1 M KCl solution.

c The data from reference 17.

**Figure 3.**
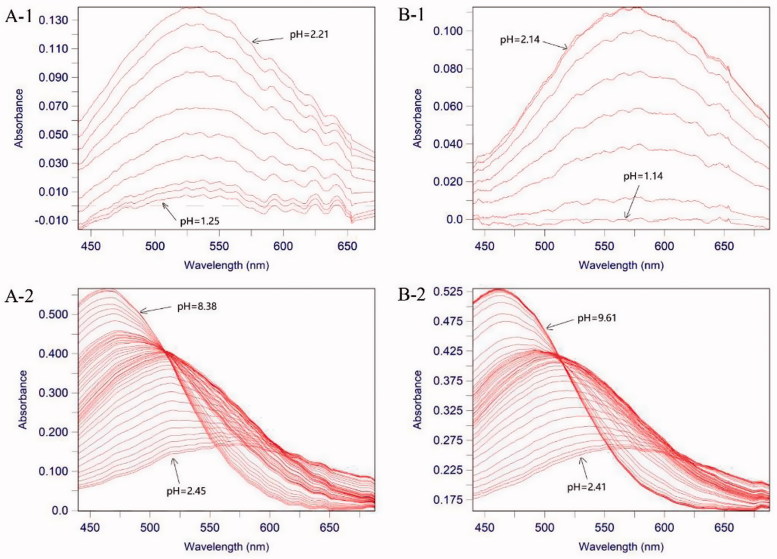
The pH-dependent UV spectra of compounds **27b** and **27f**. **A-1**. The pH-dependence of the spectrum of compound **27b** in the presence of Fe^3+^ over the pH range 1.25 and 2.21 in 0.1 M KCl at 25 °C, [Fe^3+^] = 1.0 μM, [**27b**] = 1.1 μM. **A-2**. The pH-dependence of the spectrum of compound **27b** in the presence of Fe^3+^ over the pH range 2.45 and 8.38 in 0.1 M KCl at 25 °C, [Fe^3+^] = 1.0 μM, [**27b**] = 5.0 μM. **B-1**. The pH-dependence of the spectrum of compound **27j** in the presence of Fe^3+^ over the pH range 1.14 and 2.14 in 0.1 M KCl at 25 °C, [Fe^3+^] = 1.0 μM, [**27j**] = 5.0 μM. **B-2**. The pH-dependence of the spectrum of compound **27j** in the presence of Fe^3+^ over the pH range 2.41 and 9.61 in 0.1 M KCl at 25 ^o^C, [Fe^3+^] = 1.0 μM, [**27j**] = 5.0 μM.

The stability constant of the ligand complex is one of the key parameters associated with the chelation efficiency of the ligand. The spectra of the complex of Fe^3+^ and ligand at different pH values are illustrated in Figure 3. The morphological spectrum exhibited a significant change in maximum absorption from 560 nm to 450 nm. Compounds **27a** and **27b**, displayed the most chelating activity with pFe^3+^ values of 18.9 and 19.8, respectively, which were superior to that of the positive drug deferiprone (pFe^3+^ = 17.4). Most of the compounds showed promising pFe^3+^chelating effect with pFe^3+^ values around 17.

### 3.3. Inhibition of human MAO-B

The MAO-B inhibitory properties of all the synthesised hybrids of coumarin and hydroxypyridin-4-one derivatives were tested. The compounds were first preliminarily evaluated at two concentrations, 1 and 10 μM. Compounds exhibiting significant inhibition were then submitted to the IC_50_ test. As illustrated in Table 2, when the hydroxypyridinones were introduced into the parent structure of coumarins, the obtained compounds successfully maintained the inhibitory activity against MAO-B. Most of the compounds exhibited weak anti-MAO-B effect at the concentration of 1 μM, but when the concentration reached 10 μM, the inhibitory effect was substantially over 90%. Therefore, the IC_50_ values of all compounds are below 10 μM. At the concentration of 1 μM, the inhibitory rate of compounds **27a** and **27g** exceeded 20%, while compounds **27f**, **27i,** and **27k** exceeded 30% inhibition. Compounds **27b** and **27j** were proved to be the most potent hybrids, with 54% and 62% inhibition, respectively. Compounds **27b** and **27j** were further tested for IC_50_ based on the comparison of the inhibitory activity of all compounds at two concentrations. IC_50_ values of the two compounds were 0.68 and 0.86 μM, respectively (Table 3).

**Table 2.** The MAO-B inhibitory activity of the compounds **27a-k**.

Compounds	Inhibitory rate (%)	
	1 μM	10 μM
**27a**	27.17%	96.25%
**27b**	54.02%	95.63%
**27c**	1.84%	90.98%
**27d**	9.67%	98.34%
**27e**	1.23%	95.69%
**27f**	34.48%	93.28%
**27g**	20.52%	89.46%
**27h**	13.24%	85.73%
**27i**	32.44%	94.00%
**27j**	62.43%	88.84%
**27k**	35.63%	90.52%

**Table 3.** The structures and IC_50_ values of compounds **27b** and **27j**.

Compound	Structure	IC_50_ (μM)
**27b**		0.68
**27j**		0.86
**Pargyline**		0.09

### 3.4. Structure-activity relationship discussion

In terms of the iron ion chelating activity, all target compounds demonstrated favourite iron ion chelating activity with pFe^3+^ values more than 17. When a hydroxymethyl group is introduced to the 2-position of the hydroxypyridinone, the iron chelating activity is much better. In addition, the length of the carbon chain between the hydroxypyridinone and triazole showed no obvious influence on the iron chelating effect.

The MAO-B inhibitory activity of the compounds can be well maintained by using the triazole product of Click Chemistry as the linker. Additionally, the length of the carbon chains attached to the triazole and the hydroxypyridinone have an indistinctive impact on the anti-MAO-B activity, and no significant difference in the activity of ethyl or propyl group is observed. The MAO-B inhibitory activity of most coumarin 3- or 7-substituted derivatives is significantly better than the 4-substituted compounds.

## 4. Conclusion

Hydroxypyridinone derivatives are potential agents for the AD treatment by chelating the iron ions accumulated in the brain, while coumarins also lead anti-AD candidates *via* inhibiting MAO-B. Taking the coumarin derivatives and hydroxypyridinones as the lead compounds, a series of dual-target hybrids were designed and synthesised by integrating their key pharmacophores. The targeted compounds were biologically evaluated for their iron ion chelating and MAO-B inhibitory activity. A majority of the hybrids displayed excellent iron ion chelating activity. Compounds **27b** and **27j** exhibited the most potent MAO-B inhibitory activity, and IC_50_ values of the two compounds were 0.68 and 0.86 μM, respectively.

In summary, the hybrids of coumarin derivatives and hydroxypyridinones were proved to display both potent iron ion chelating effect and MAO-B inhibitory activity. These dual-target compounds have the potential to demonstrate *in vivo* anti-AD activity. These results also proved that developing dual-target agents is a possible strategy for the treatment of AD.

## Supplementary Material

Supplemental Material
