# Supplementary material for: Dual-target anti-Alzheimer’s disease agents with both iron ion chelating and monoamine oxidase-B inhibitory activity
Source: J Enzyme Inhib Med Chem. 2019 Aug 16;34(1):1489–97. doi: 10.1080/14756366.2019.1634703 (PMC6713216; doi:10.1080/14756366.2019.1634703)
Supplement: Supplemental Material [file IENZ_A_1634703_SM8196.pdf]

Dual-target anti-Alzheimer's disease agents with both iron ion chelating and monoamine oxidase-B inhibitory activity

Zhisheng Mi [a, #], Bing Gan [a, b, #], Sihang Yu [c, #], Jianan Guo [a], Changjun Zhang [a], Xiaoying Jiang [a], Tao Zhou[d], Jing Su [d, *], Renren Bai [a, *], Yuanyuan Xie [a, e, *]

[a]College of Pharmaceutical Science, Zhejiang University of Technology, Hangzhou, China

[b]Guiyang Institute for Food and Drug Control, Guiyang, China

[c]Department of Pathophysiology, College of Basic Medical Sciences, Jilin University, Changchun, China

[d]School of Food Science and Biotechnology, Zhejiang Gongshang University, Hangzhou, China

[e]Collaborative Innovation Center of Yangtze River Delta Region Green Pharmaceuticals, Zhejiang University of Technology, Hangzhou, China

[#]These authors contributed equally to this work

Correspondence:

Dr. Yuanyuan Xie, Key Laboratory for Green Pharmaceutical Technologies and Related Equipment of Ministry of Education, College of Pharmaceutical Sciences, Zhejiang University of Technology, Hangzhou 310014, China. Email: xyycz@zjut.edu.cn

Dr. Renren Bai, College of Pharmaceutical Sciences, Zhejiang University of Technology, Hangzhou 310014, China. Email: renrenbai@zjut.edu.cn

Dr. Jing Su, Department of Pathophysiology, College of Basic Medical Sciences, Jilin University, Changchun, China. Email: sujing@jlu.edu.cn

27a

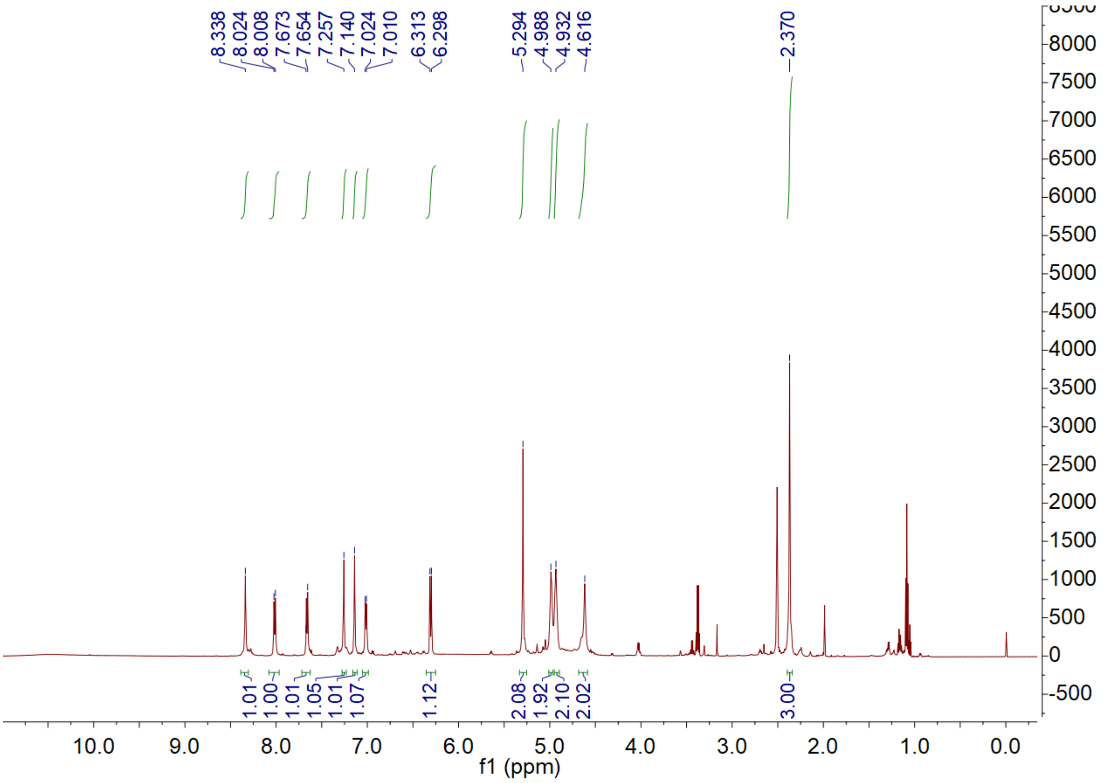
8.338
8.024
8.008
7.673
7.654
7.257
7.140
7.024
7.010
6.313
6.298
5.294
4.988
4.932
4.616
2.370
1.01
1.00
1.01
1.05
1.01
1.07
1.12
2.08
1.92
2.10
2.02
3.00
f1 (ppm)

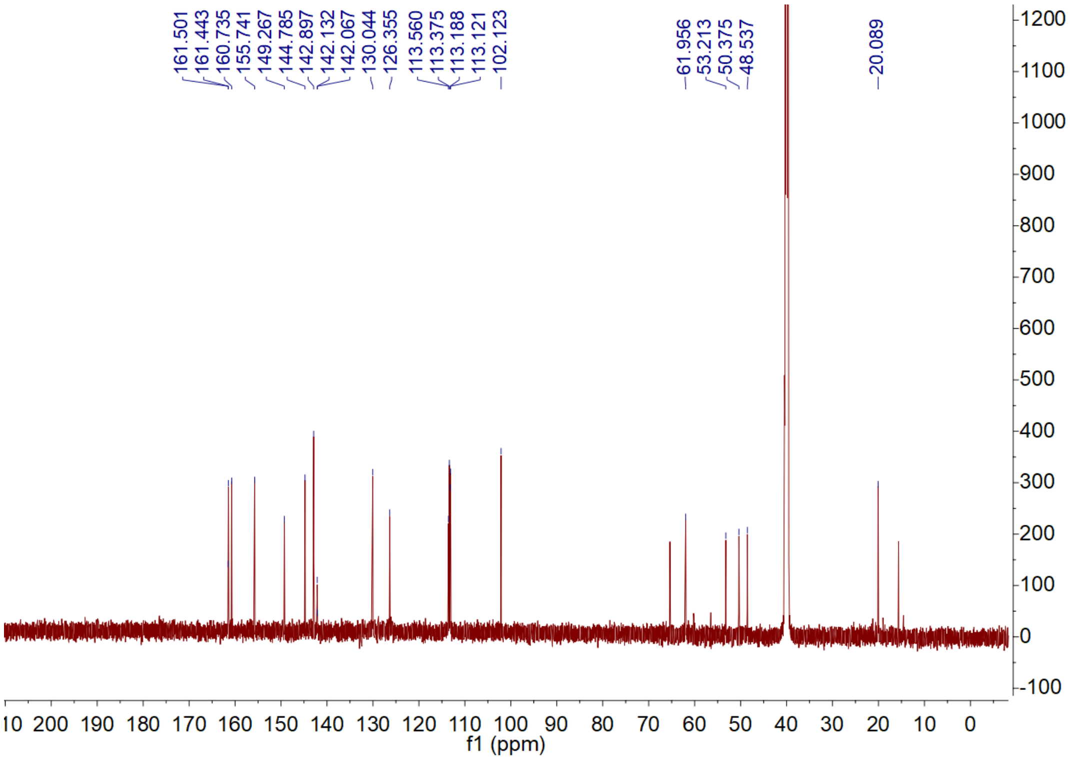
161.501
161.443
160.735
155.741
149.267
144.785
142.897
142.132
142.067
130.044
126.355
113.560
113.375
113.188
113.121
102.123
61.956
53.213
50.375
48.537
20.089
f1 (ppm)

27b

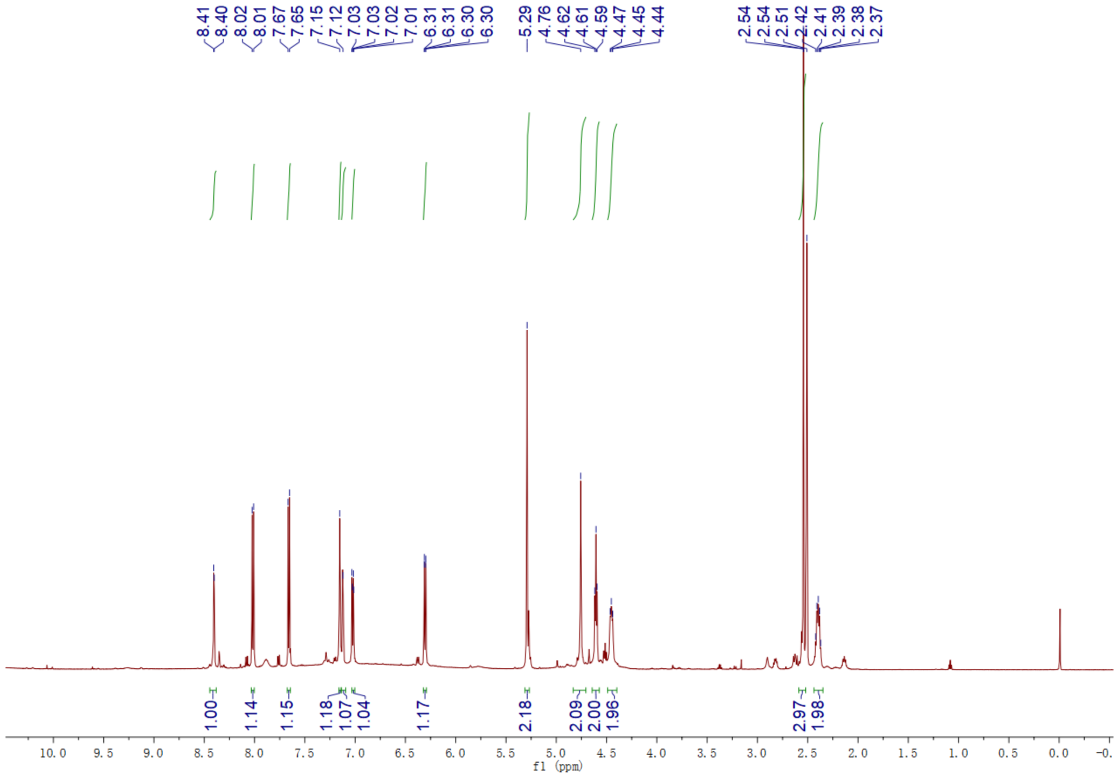

8.41
8.40
8.02
8.01
7.67
7.65
7.15
7.12
7.03
7.03
7.02
7.01
6.31
6.31
6.30
6.30
5.29
4.76
4.62
4.61
4.59
4.47
4.45
4.44
2.54
2.54
2.51
2.42
2.41
2.39
2.38
2.37
1.00
1.14
1.15
1.18
1.07
1.04
1.17
2.18
2.09
2.00
1.96
2.97
1.98
f1 (ppm)

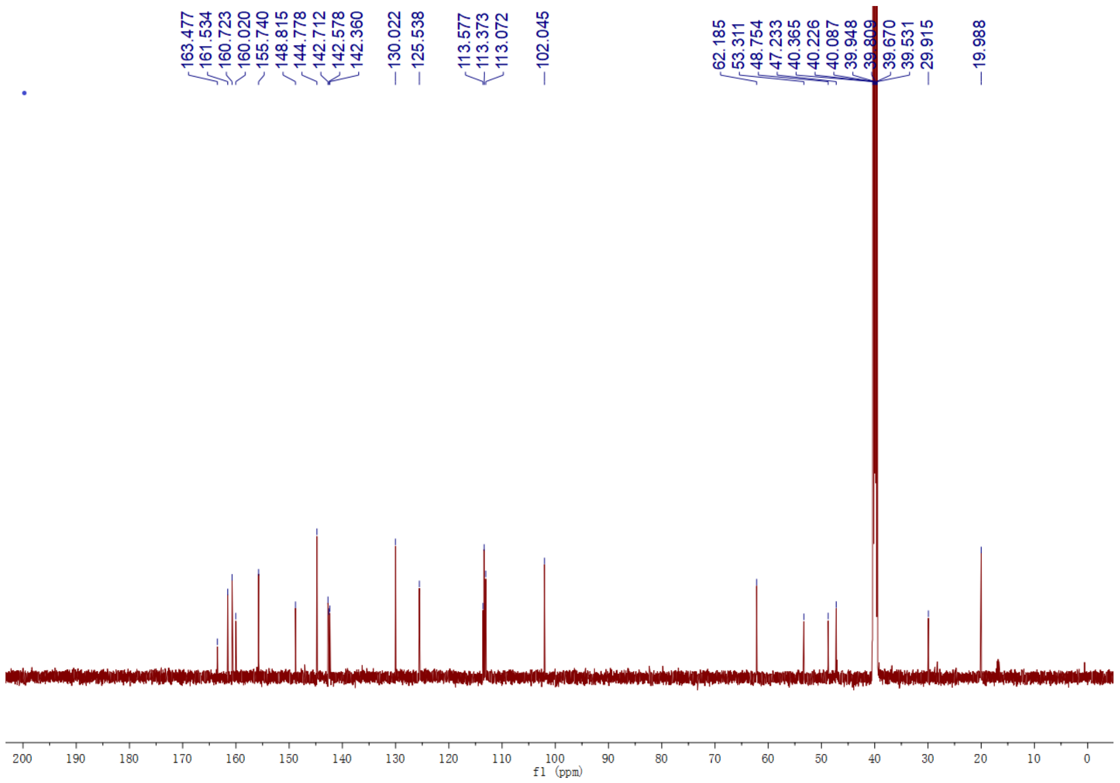

163.477
161.534
160.723
160.020
155.740
148.815
144.778
142.712
142.578
142.360
130.022
125.538
113.577
113.373
113.072
102.045
62.185
53.311
48.754
47.233
40.365
40.226
40.087
39.948
39.809
39.670
39.531
29.915
19.988
f1 (ppm)

27c

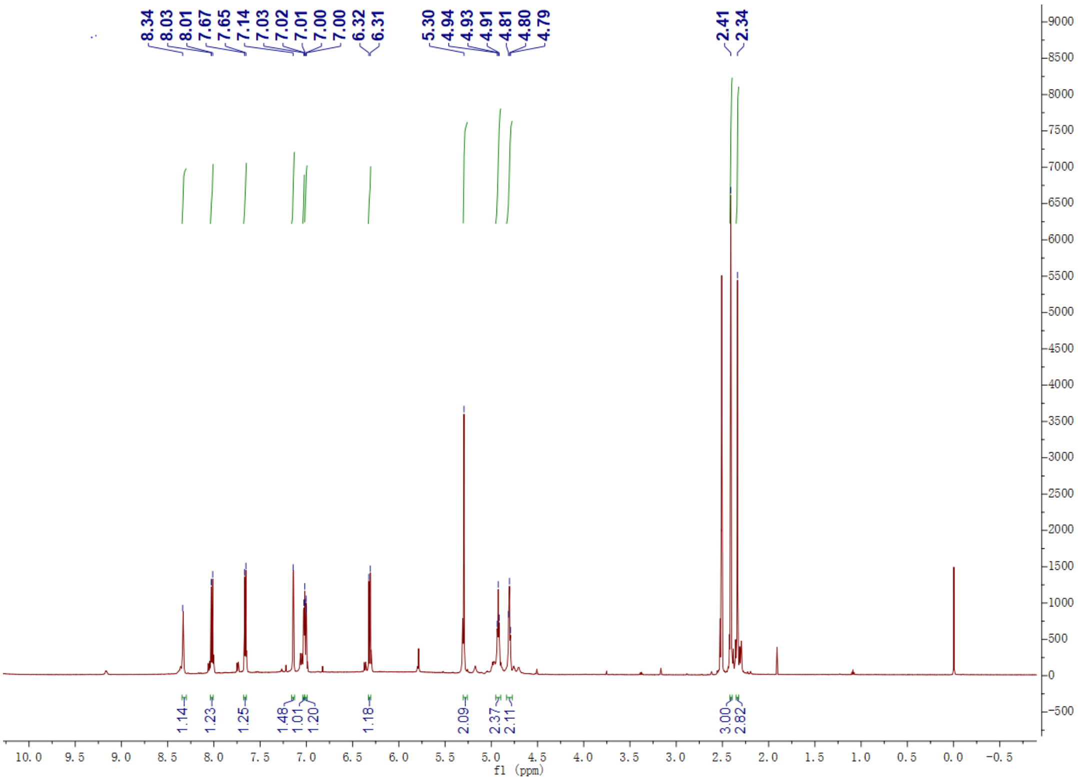
8.34
8.03
8.01
7.67
7.65
7.14
7.03
7.02
7.01
7.00
7.00
6.32
6.31
5.30
4.94
4.93
4.91
4.81
4.80
4.79
2.41
2.34
1.14
1.23
1.25
1.48
1.01
1.20
1.18
2.09
2.37
2.11
3.00
2.82
f1 (ppm)

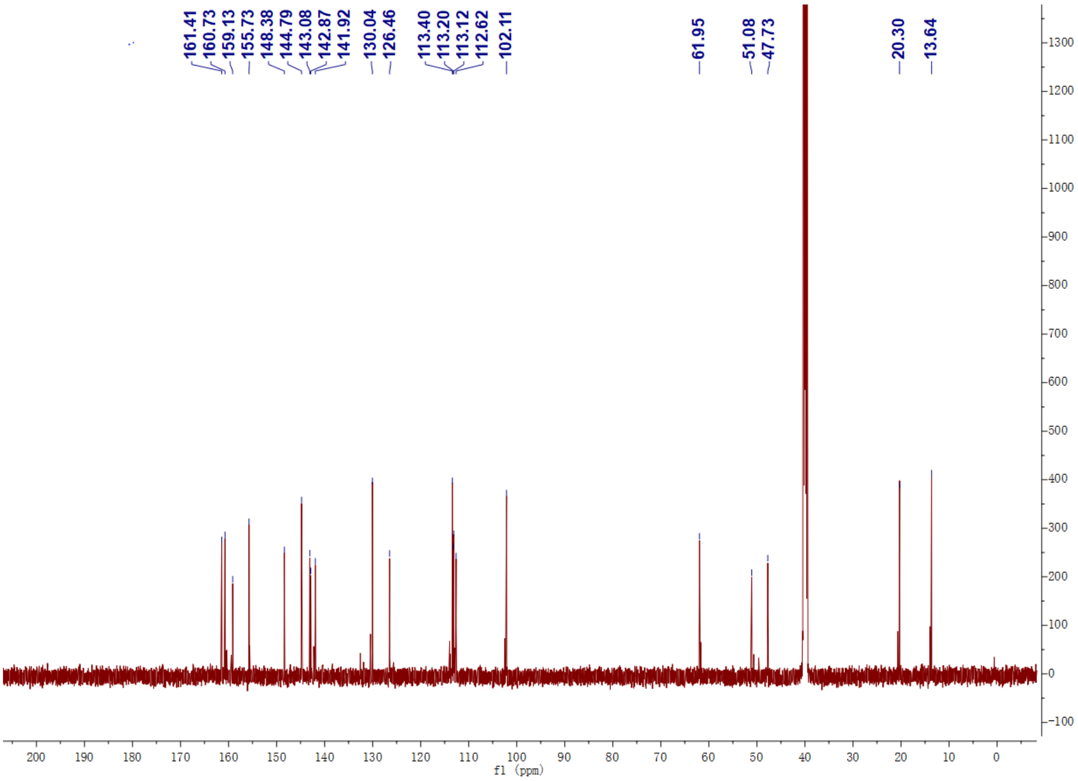
161.41
160.73
159.13
155.73
148.38
144.79
143.08
142.87
141.92
130.04
126.46
113.40
113.20
113.12
112.62
102.11
61.95
51.08
47.73
20.30
13.64
f1 (ppm)

27d

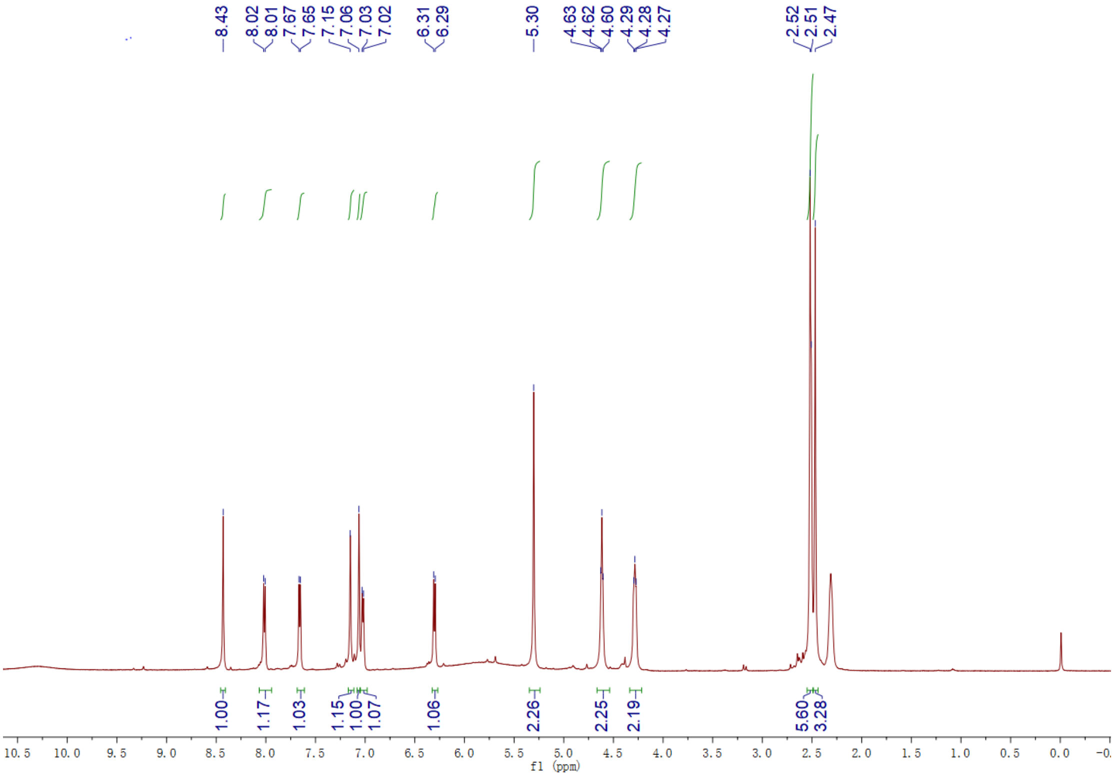

8.43
8.02
8.01
7.67
7.65
7.15
7.06
7.03
7.02
6.31
6.29
5.30
4.63
4.62
4.60
4.29
4.28
4.27
2.52
2.51
2.47
1.00
1.17
1.03
1.15
1.00
1.07
1.06
2.26
2.25
2.19
5.60
3.28
f1 (ppm)

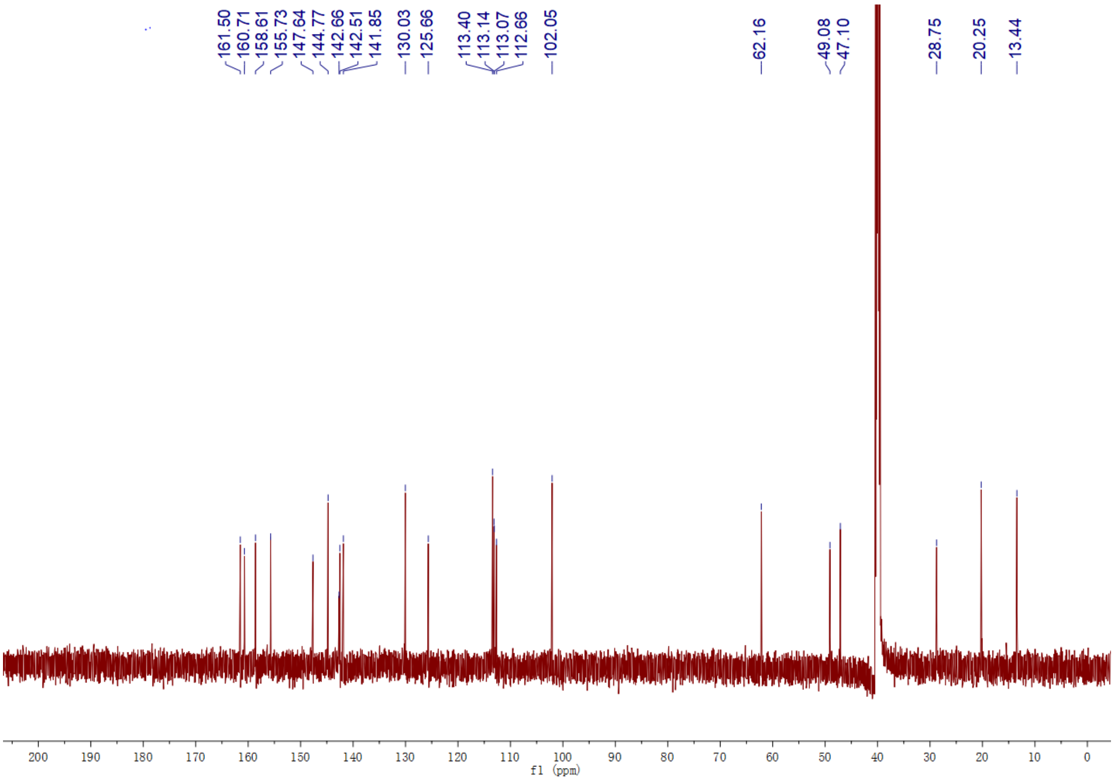

161.50
160.71
158.61
155.73
147.64
144.77
142.66
142.51
141.85
130.03
125.66
113.40
113.14
113.07
112.66
102.05
62.16
49.08
47.10
28.75
20.25
13.44
f1 (ppm)

27e

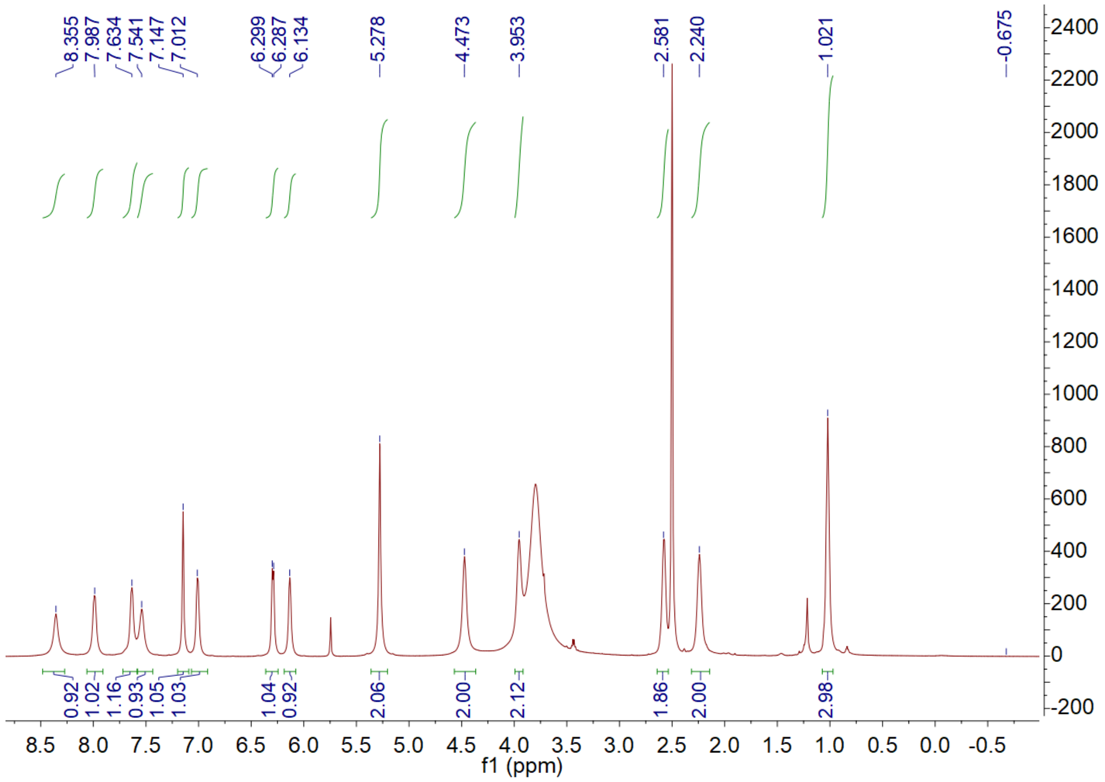
8.355
7.987
7.634
7.541
7.147
7.012
6.299
6.287
6.134
5.278
4.473
3.953
2.581
2.240
1.021
-0.675
0.92
1.02
1.16
0.93
1.05
1.03
1.04
0.92
2.06
2.00
2.12
1.86
2.00
2.98
f1 (ppm)

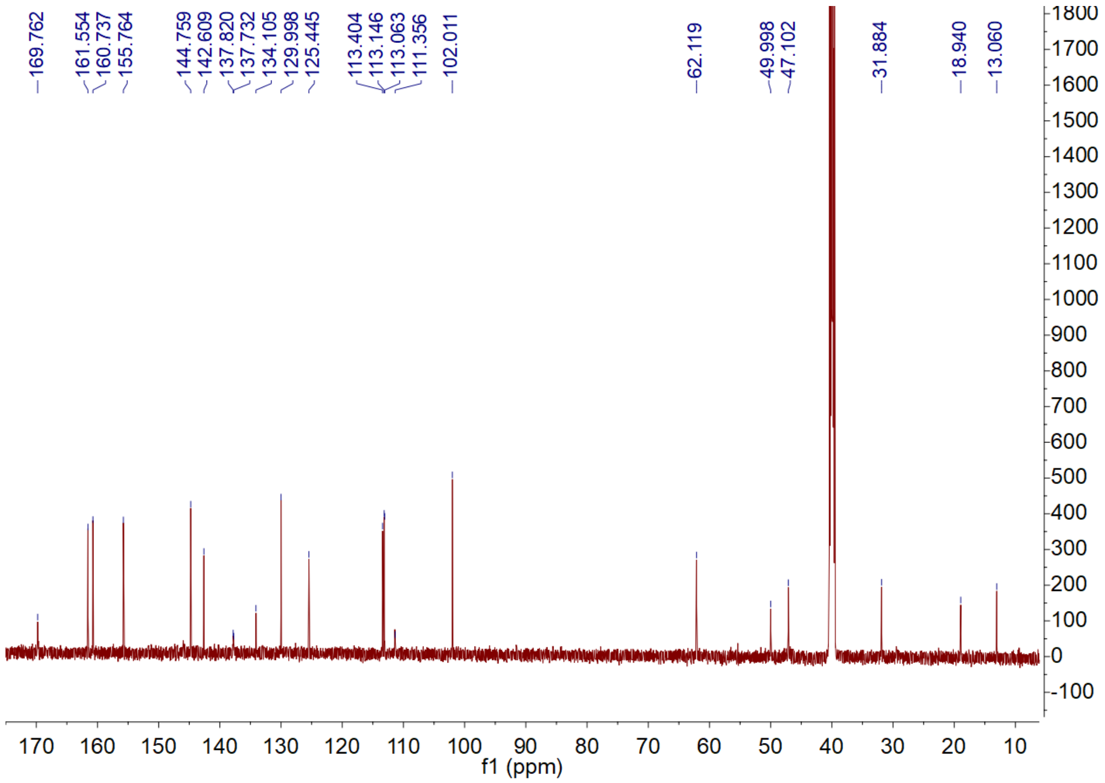
169.762
161.554
160.737
155.764
144.759
142.609
137.820
137.732
134.105
129.998
125.445
113.404
113.146
113.063
111.356
102.011
62.119
49.998
47.102
31.884
18.940
13.060
f1 (ppm)

27f

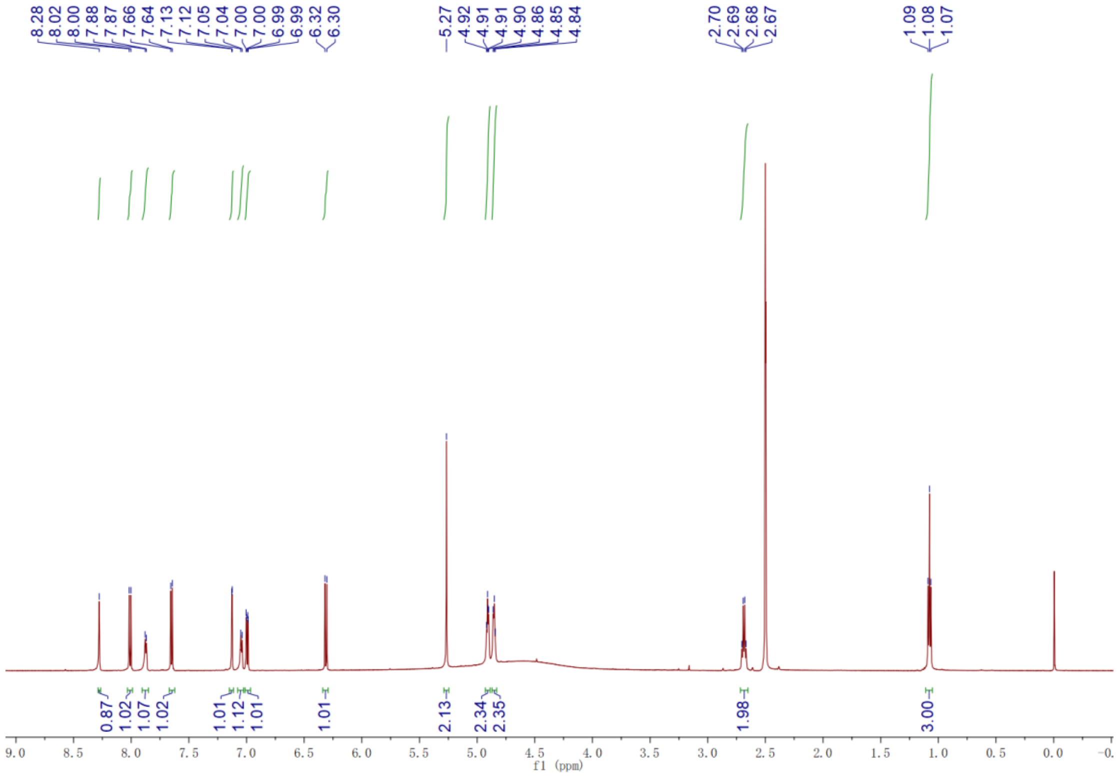

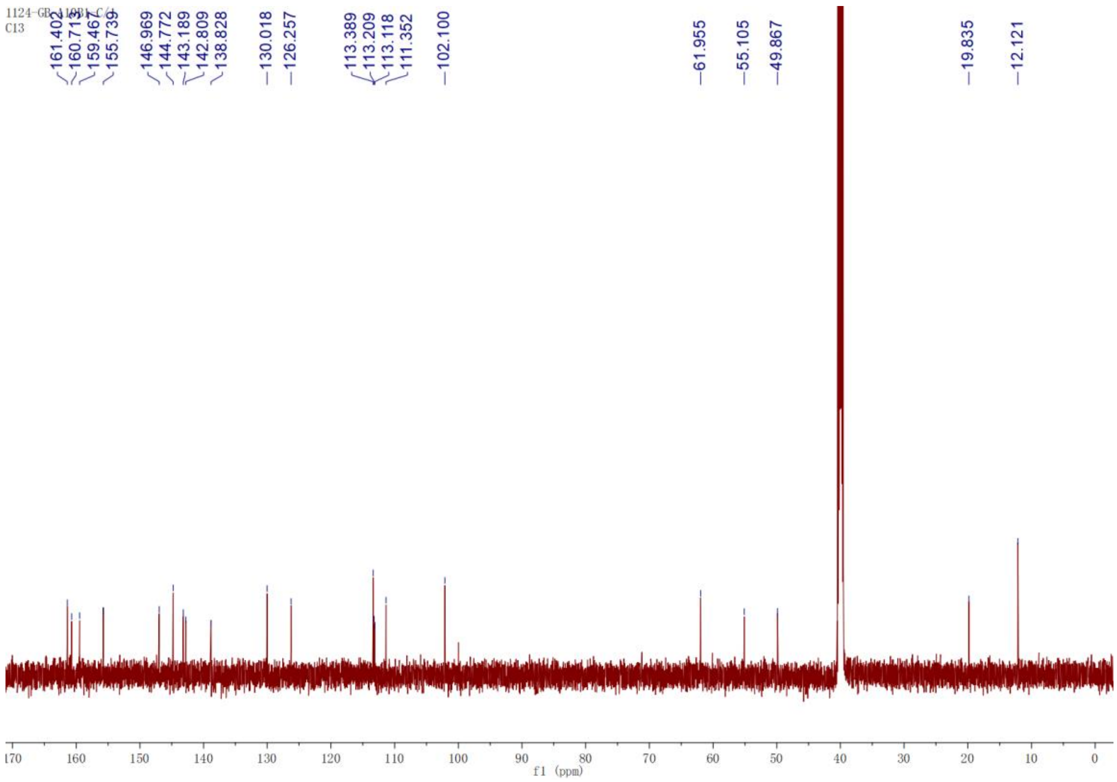

27g

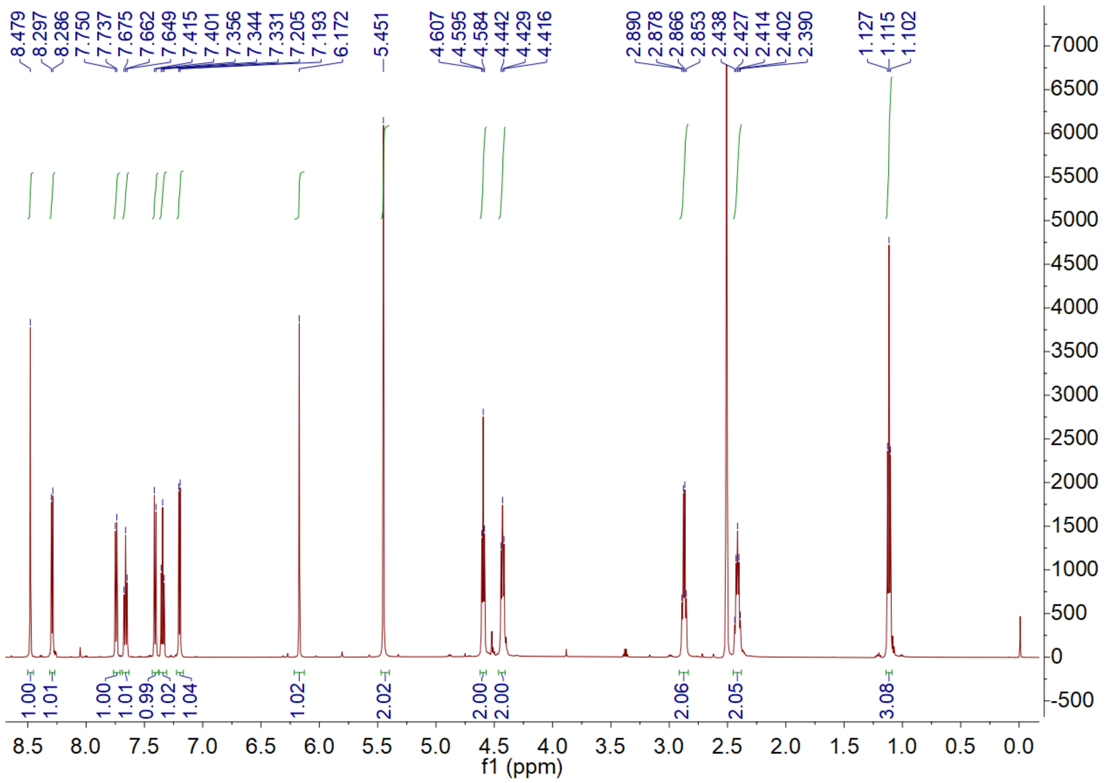

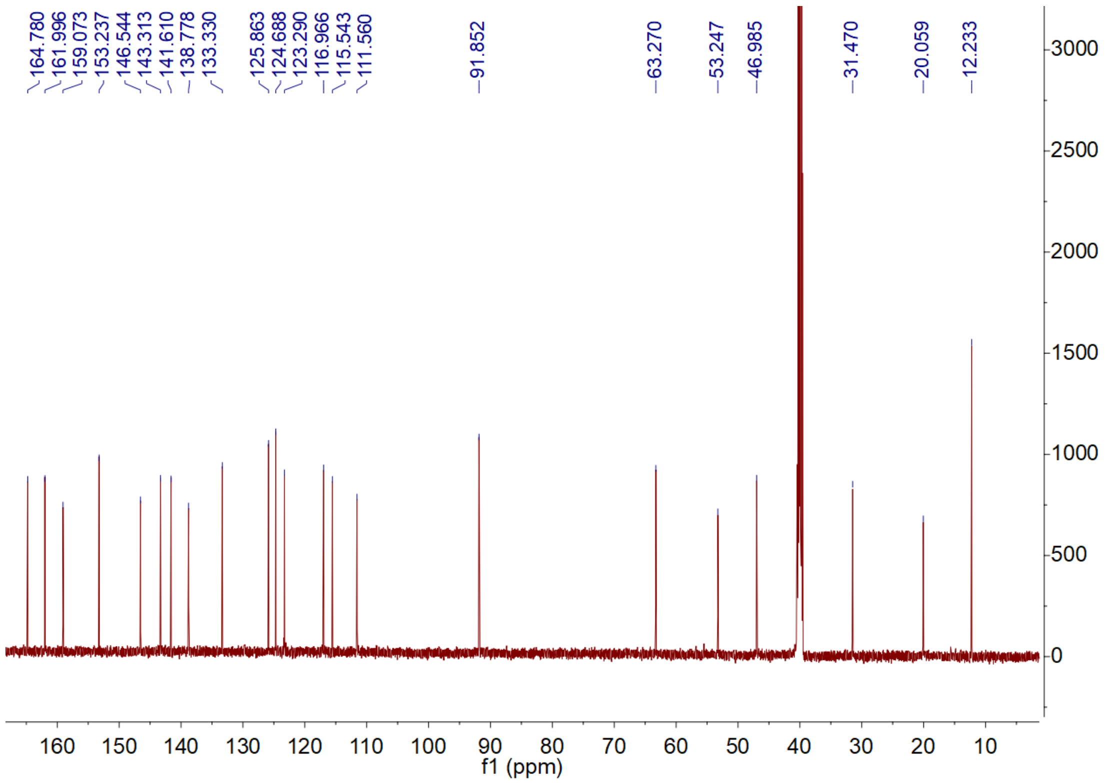

27h

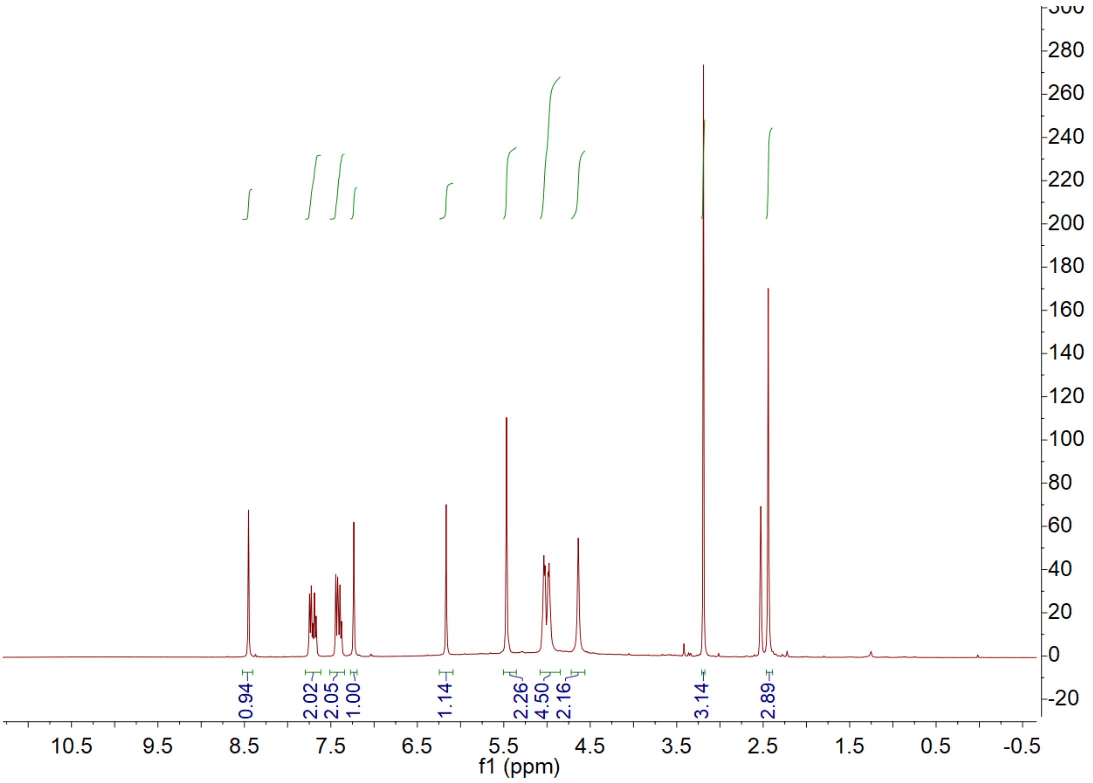
0.94
2.02
2.05
1.00
1.14
2.26
4.50
2.16
3.14
2.89
f1 (ppm)

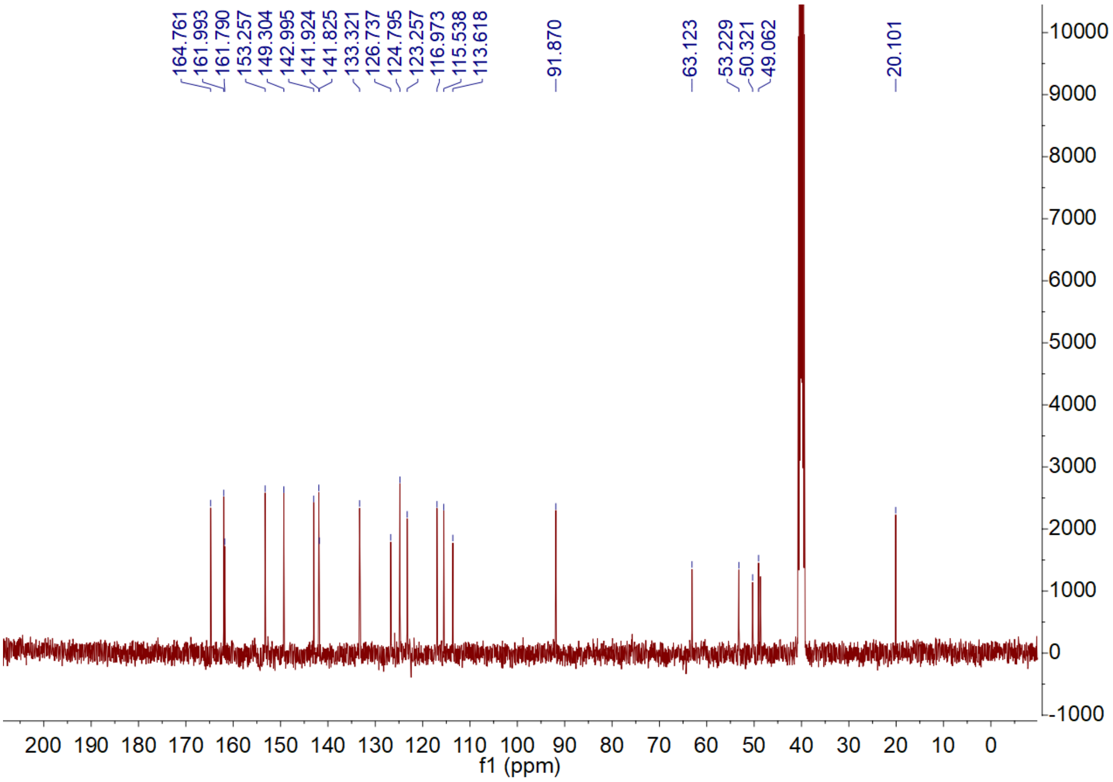
164.761
161.993
161.790
153.257
149.304
142.995
141.924
141.825
133.321
126.737
124.795
123.257
116.973
115.538
113.618
91.870
63.123
53.229
50.321
49.062
20.101
f1 (ppm)

27i

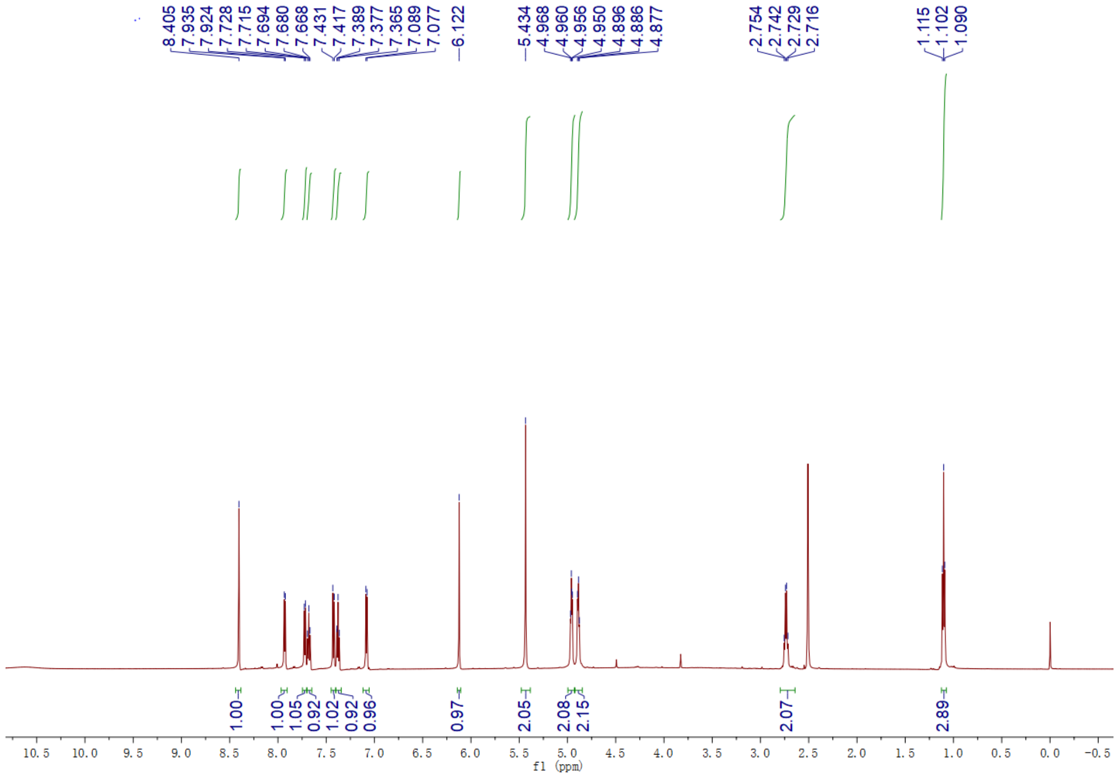
8.405
7.935
7.924
7.728
7.715
7.694
7.680
7.668
7.431
7.417
7.389
7.377
7.365
7.089
7.077
6.122
5.434
4.968
4.960
4.956
4.950
4.896
4.886
4.877
2.754
2.742
2.729
2.716
1.115
1.102
1.090
1.00
1.00
1.05
0.92
1.02
0.92
0.96
0.97
2.05
2.08
2.15
2.07
2.89
f1 (ppm)

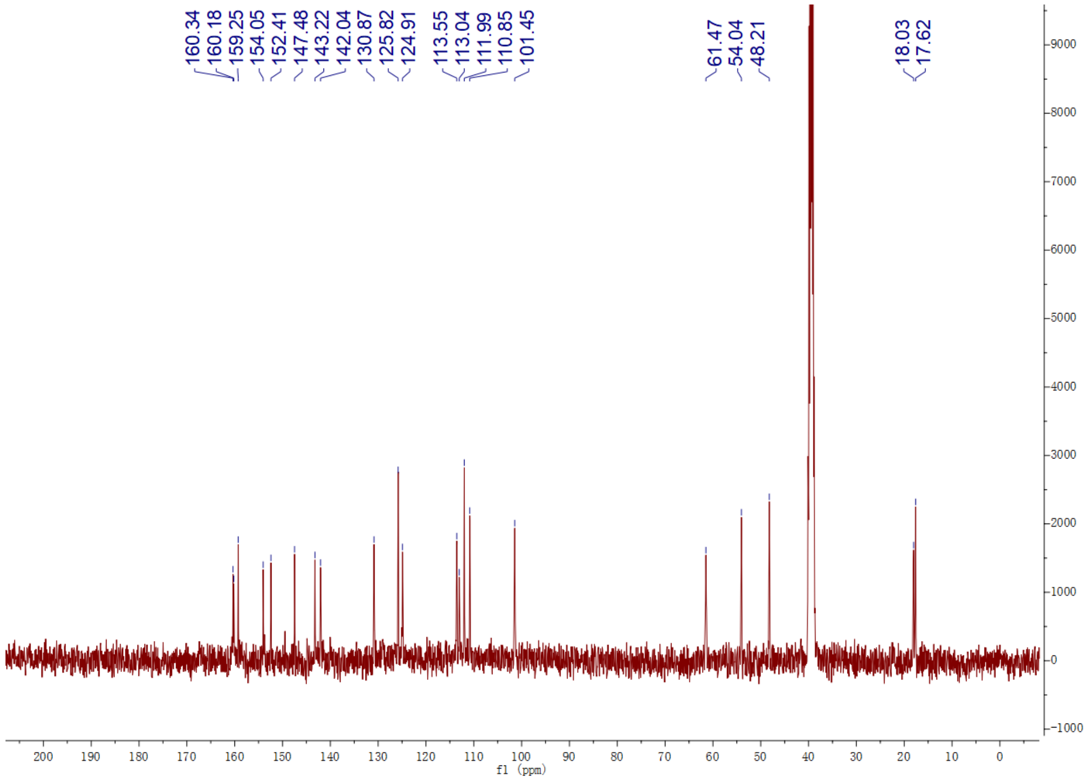
160.34
160.18
159.25
154.05
152.41
147.48
143.22
142.04
130.87
125.82
124.91
113.55
113.04
111.99
110.85
101.45
61.47
54.04
48.21
18.03
17.62
f1 (ppm)

27j

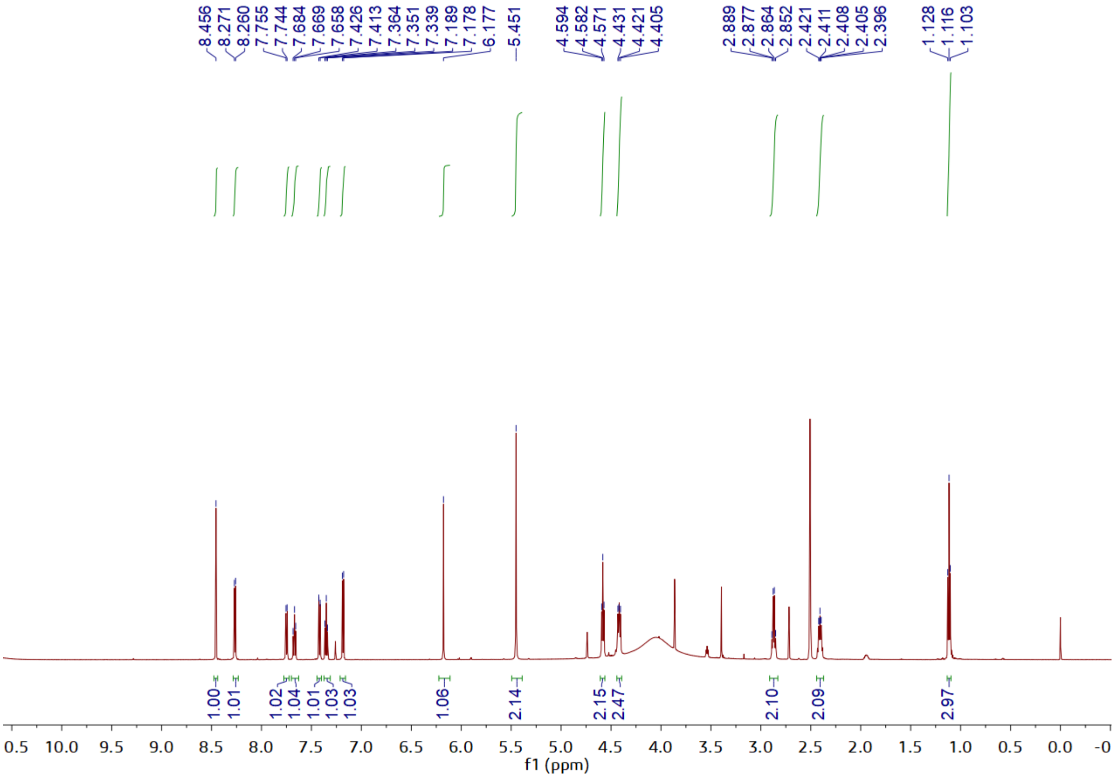
8.456
8.271
8.260
7.755
7.744
7.684
7.669
7.658
7.426
7.413
7.364
7.351
7.339
7.189
7.178
6.177
5.451
4.594
4.582
4.571
4.431
4.421
4.405
2.889
2.877
2.864
2.852
2.421
2.411
2.408
2.405
2.396
1.128
1.116
1.103
1.00
1.01
1.02
1.04
1.01
1.03
1.03
1.06
2.14
2.15
2.47
2.10
2.09
2.97
f1 (ppm)

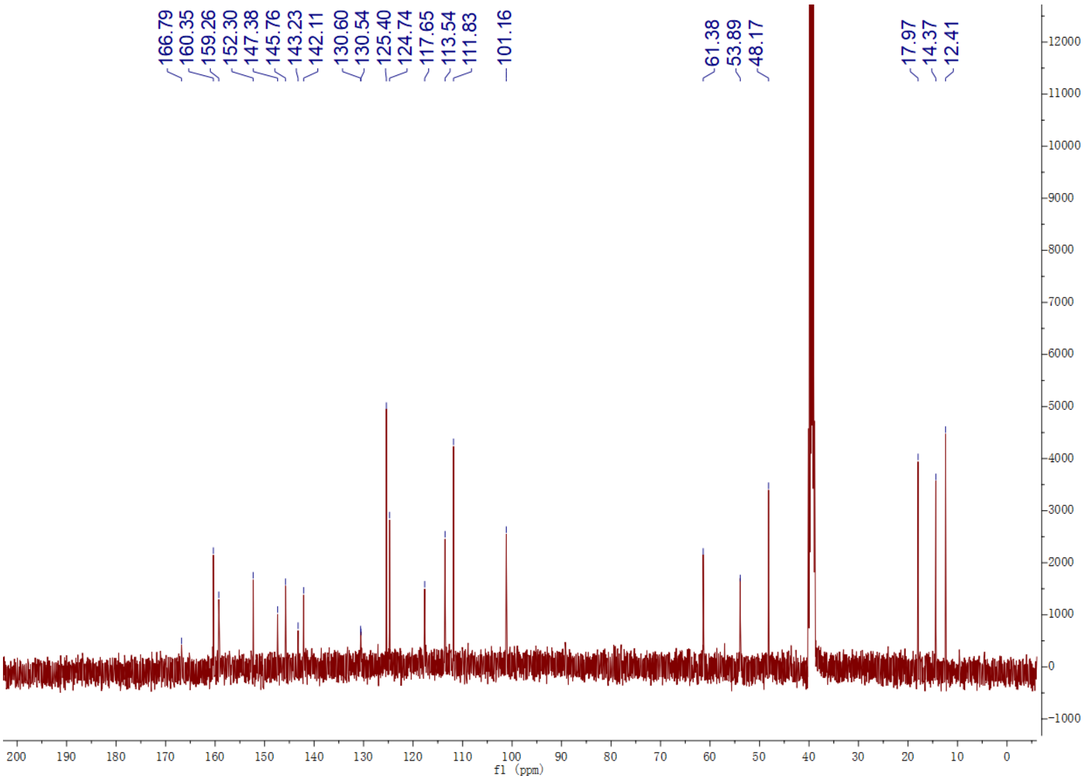
166.79
160.35
159.26
152.30
147.38
145.76
143.23
142.11
130.60
130.54
125.40
124.74
117.65
113.54
111.83
101.16
61.38
53.89
48.17
17.97
14.37
12.41
f1 (ppm)

27k

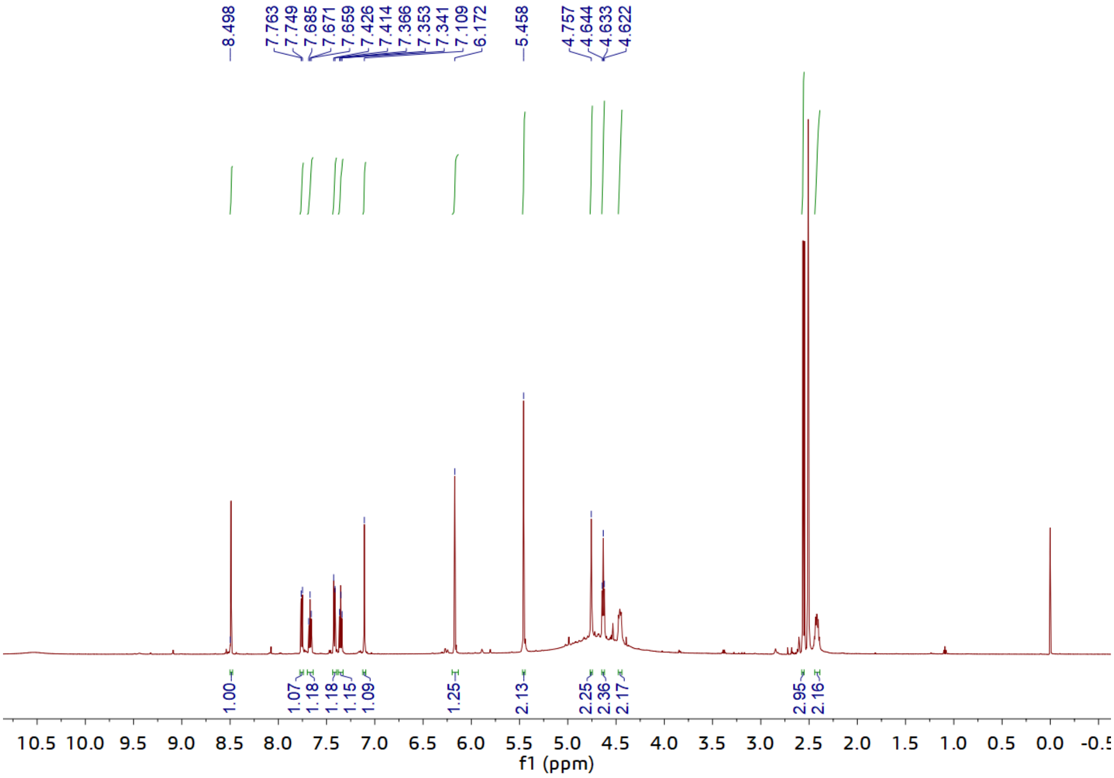

8.498
7.763
7.749
7.685
7.671
7.659
7.426
7.414
7.366
7.353
7.341
7.109
6.172
5.458
4.757
4.644
4.633
4.622
1.00
1.07
1.18
1.18
1.15
1.09
1.25
2.13
2.25
2.36
2.17
2.95
2.16
f1 (ppm)

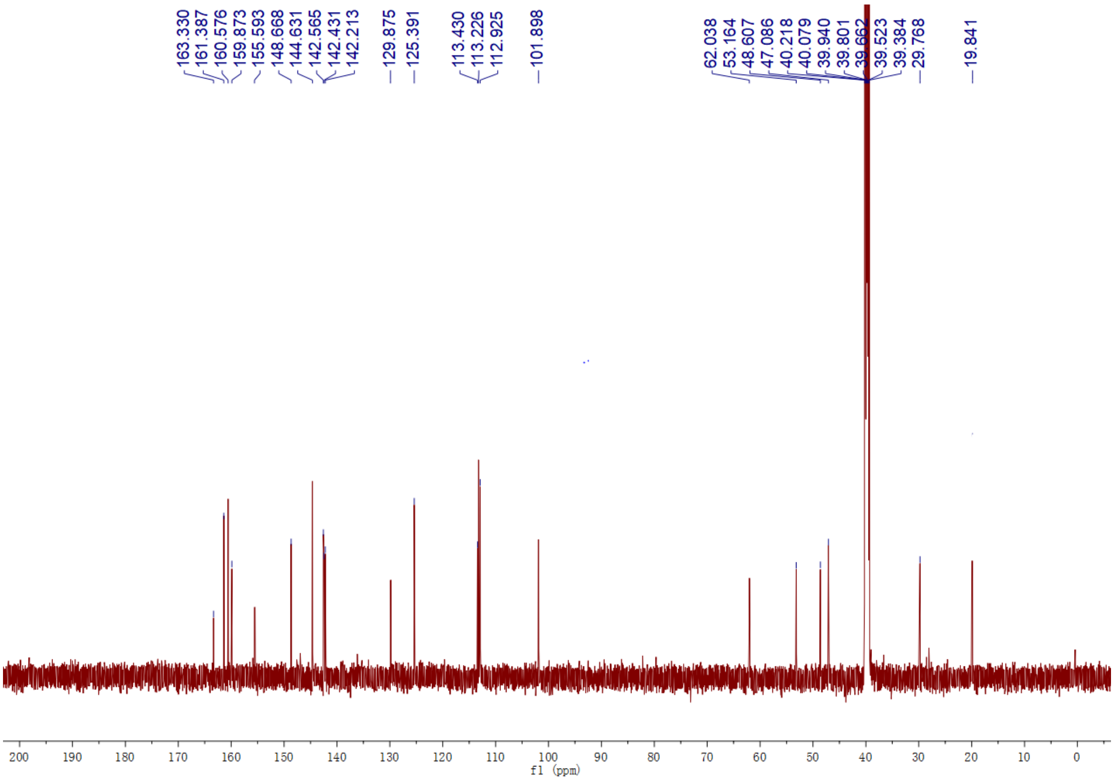

163.330
161.387
160.576
159.873
155.593
148.668
144.631
142.565
142.431
142.213
129.875
125.391
113.430
113.226
112.925
101.898
62.038
53.164
48.607
47.086
40.218
40.079
39.940
39.801
39.662
39.523
39.384
29.768
19.841
f1 (ppm)
